# Emerging Magnetic Fabrication Technologies Provide Controllable Hierarchically‐Structured Biomaterials and Stimulus Response for Biomedical Applications

**DOI:** 10.1002/advs.202202278

**Published:** 2022-10-13

**Authors:** Jacek K. Wychowaniec, Dermot F. Brougham

**Affiliations:** ^1^ School of Chemistry University College Dublin Belfield Dublin 4 Ireland; ^2^ AO Research Institute Davos Clavadelerstrasse 8 Davos 7270 Switzerland

**Keywords:** advanced manufacturing technologies, controlled release, magnetic hydrogels, magnetic hyperthermia, magnetic patterning, soft robotics

## Abstract

Multifunctional nanocomposites which exhibit well‐defined physical properties and encode spatiotemporally‐controlled responses are emerging as components for advanced responsive systems. For biomedical applications magnetic nanocomposite materials have attracted significant attention due to their ability to respond to spatially and temporally varying magnetic fields. The current state‐of‐the‐art in development and fabrication of magnetic hydrogels toward biomedical applications is described. There is accelerating progress in the field due to advances in manufacturing capabilities. Three categories can be identified: i) Magnetic hydrogelation, DC magnetic fields are used during solidification/gelation for aligning particles; ii) additive manufacturing of magnetic materials, 3D printing technologies are used to develop spatially‐encoded magnetic properties, and more recently; iii) magnetic additive manufacturing, magnetic responses are applied during the printing process to develop increasingly complex structural arrangement that may recapitulate anisotropic tissue structure and function. The magnetic responsiveness of conventionally and additively manufactured magnetic hydrogels are described along with recent advances in soft magnetic robotics, and the categorization is related to final architecture and emergent properties. Future challenges and opportunities, including the anticipated role of combinatorial approaches in developing 4D‐responsive functional materials for tackling long‐standing problems in biomedicine including production of 3D‐specified responsive cell scaffolds are discussed.

## Graphical abstract

Classification of fabrication technologies for magnetic materials is provided including: i) Magnetic hydrogelation; ii) additive manufacturing of magnetic materials, and; iii) the emerging field magnetic additive manufacturing. The magnetic responsiveness of conventionally and additively manufactured magnetic hydrogels is described along with recent advances toward biomedical applications.

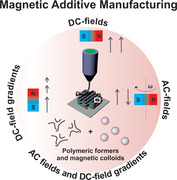

## 1 Introduction

**Figure 1:**
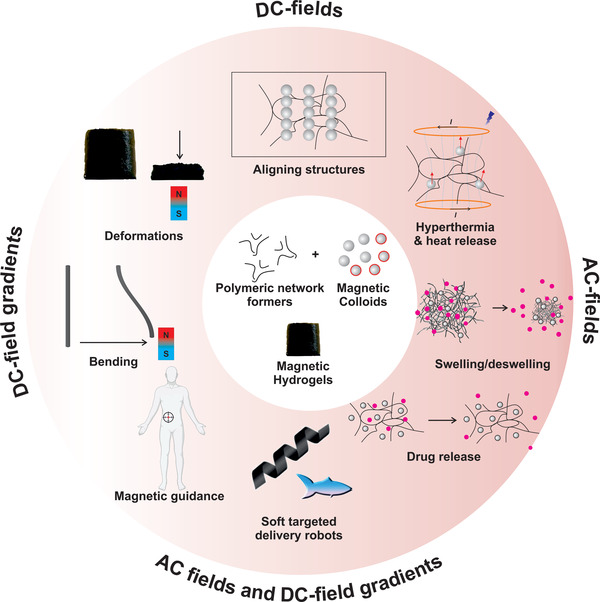
Scheme depicting the different types of responses obtained from magnetic nanocomposite hydrogels (Mag‐Gels) on exposure to DC‐ (static) or AC‐ (alternating) magnetic fields. These stimuli can be combined to generate targeted therapies, for instance using field‐gradients to guide magnetic nano‐agents that release cargo on AC‐field stimulus.

Researchers working across multiple fields are currently pursuing multifunctional nanocomposite systems that can provide well‐defined reproducible responses in both spatial and temporal domains for biomedical applications, such as remotely controlled mechano‐transduction for precise tuning of cellular processes.^[^ ^1^ ^]^ In this regard magnetic nanocomposite materials have attracted significant attention due to their unparalleled ability to provide programmed functional responses on application of magnetic forces, for instance, time‐dependent deformations for use in transport and release.^[^ ^2^ ^]^ In particular, magnetic structures respond in a variety of ways to static‐, varying‐, and AC‐field stimulus (**Figure** **1**) which can be actuated without contact as the applied fields permeate most materials including the human body, enabling precise spatially‐specified response and (safe) directional movement.^[^ ^3^ ^]^

To form functional magnetically responsive nanocomposites, two components are required: i) Suspensions of magnetic nanoparticles or nanoparticle clusters/assemblies (i.e. a magnetic fraction); and ii) a matrix, typically made of responsive biocompatible polymers (Figure 1).^[^ ^4^ ^]^ Guidelines for formation of magnetic nanocomposite hydrogels using different matrices were previously given by Thevenot et al.,^[^ ^4^ ^]^ and more recently by Li et al.,^[^ ^5^ ^]^ providing a classification by the type of magnetic response for nanocomposite hydrogels fabricated using conventional techniques only.

Over the last decade a multitude of additive manufacturing (AM) technologies have been used in the field, often with the aim of advancing personalized medicine, for example, by providing possibilities for generating patient‐specific organoids or for stratification of responders before drug treatment.^[^ ^6^ ^]^ Some next‐generation multi‐responsive (including magnetically) and hierarchically organized bulk nanocomposites, as well as soft robot‐like devices, for example, for cell manipulation, have emerged.^[^ ^7^ ^]^ Structuring biocompatible hydrogels across multiple length scales, from nano‐ up to macro‐, remains a goal and a guiding principle in developing biomaterials that mimic natural tissue organization and responsiveness.^[^ ^8^ ^]^

Some recent reviews described use of magnetic nanocomposite inks or application of magnetic responses during manufacturing,^[^ ^9^ ^]^ however, these only briefly mentioned exemplar studies and did not provide a complete view of the field. Magnetically‐responsive nanocomposite hydrogels were previously categorized as ink components used for extrusion‐based AM.^[^ ^10^ ^]^ Shape changing materials^[^ ^11^ ^]^ and magnetically driven micro‐ and nanorobots^[^ ^12^ ^]^ have also been recently reviewed. One application of magnetic nanocomposites; drug release by AC‐field induced hyperthermia (Figure 1) using thermoresponsive polymers, has also been described.^[^ ^13^ ^]^ To the best of our knowledge, this is the first overview to include categorization of soft magnetic materials (including the underlying interactions between magnetic and non‐magnetic components), the possible magnetic manipulations (forces), and the additive manufacturing techniques and how these can be advantageously combined toward achieving functional biomaterials.

In Subsections 1.1–1.4 the technical aspects of formulating magnetic inks, of magnetic field delivery during fabrication, and some of the considerations for bio‐applications are described. In Section 2 the emerging magnetic hydrogel fabrication techniques; conventional, advanced manufacturing, and magnetic advanced manufacturing are described. Detailed analysis of the biomedical applications of these soft, patterned materials, and the challenges in preparing them follows in Section 3.

### 1.1 Magnetic Responses of Suspensions of Magnetic Nanoparticles

Before considering soft magnetic nanocomposites, the responses of magnetic nanoparticle suspensions on application of different magnetic stimuli are described.^[^ ^9^ ^]^ Suspensions of magnetic iron‐oxide nanoparticles (MNPs) and magnetic nanoparticle assemblies or clusters (MNPCs) are used in a wide range of biomedical applications from cancer treatment, to magnetic resonance imaging (MRI) and in vivo stem cell tracking/capture/reprogramming.^[^ ^14^ ^]^ For sub‐20 nm MNPs rapid reorientation of independent magnetic moments (superparamagnetism) can afford particle size‐ and concentration‐tuneable magnetic responses. The absence of bulk magnetization when the field is removed enhances formulation stability, a critical aspect for nanocomposite processing/printing.^[^ ^15^ ^]^

Magnetic responses commonly studied for the suspensions include: i) Alignment of particle moments on exposure to static‐fields (low Tesla range) which can induced particle assembly into chains, ii) translational forces on particles^[^ ^16^ ^]^ on exposure to strong field‐gradients (static‐fields, of magnetic force B∇B 10–500 T^2^ m^−1^) resulting in directed movement; iii) rapid heating^[^ ^17^ ^]^ or particle re‐orientation^[^ ^18^ ^]^ on exposure to AC‐magnetic fields (*H*_AC_, 2–10 kA m^−1^, *ν*_AC_ 100–900 kHz). Note that the individual fields of magnetic MNP formation,^[^ ^19^ ^]^ assembly in colloidal suspension,^[^ ^16^ ^]^ and applications in stem cell tracking for regenerative medicine have also recently been reviewed.^[^ ^20^ ^]^ Hence we will not touch on these topics except to outline some of the key physical concepts underlying the magnetic responses. The main responses include are described below.

#### 1.1.1 AC‐Field Induced Hyperthermia

The heating efficiency of particles under adiabatic conditions is defined by the specific absorption rate (SAR, units W g^−1^) of AC‐field energy as;

(1) $$\mathrm{SAR}=\frac{CV_{s}}{m_{\mathrm{Fe}}}\times {\left[\frac{dT}{dt}\right]}_{t=0}$$

where *C* is the volumetric heat capacity (J mL^−1^ °C^−1^) of the medium, *V*_s_ the volume of the sample (mL), *m*_Fe_ the mass of iron (in g) and ${\left[\frac{dT}{dt}\right]}_{t=0}$ (°C s^−1^) defines an initial “linear” response of the temperature‐time response, extracted from the linear term of at least 4th order polynomial fit, a commonly accepted approach.^[^ ^21^ ^]^ SAR values are frequency and field strength dependent, but are almost invariably reported for a wide range of conditions. The intrinsic loss power (ILP, units W m^2^ g^−1^ kA^−2^ kHz^−1^) is defined as:

(2) $$\mathrm{ILP}=\frac{\mathrm{SAR}}{\nu_{\mathrm{AC}}\times H_{\mathrm{AC}}^{2}}$$

This scales the response, and is appropriate within the linear response regime,^[^ ^15^ ^]^ enabling direct comparisons of hyperthermic efficacy for measurements performed under different conditions. However it is not always quoted in the literature.

Biomedical applications of hyperthermia include: i) Direct injection of concentrated suspensions for cancer ablation at the site, usually as a palliative treatment, and; ii) intravenous injection of long‐circulating particles usually for tumor deposition by enhanced permeability and retention, effects, and; iii) delivery of biomolecules and drugs.^[^ ^14^, ^22^ ^]^ There is a very substantial literature on maximizing SAR values for suspensions which is key for all approaches, but complications in its measurement arise due to both non‐adiabaticity of real systems and colloidal instability, both of which are impacted by the high concentrations used. Even if the suspensions are stable any aggregation results in inter‐particle dipolar interactions that slow moment dynamics, usually reducing hyperthermic efficiency.^[^ ^23^ ^]^ Hence high particle load, excellent colloidal stability and high hyperthermic efficiency are critical competing factors that determine the thermal dose deliverable, with SAR values in the range of 1–3 kW g^−1^ reported for suspensions in recent years. Developing reproducible and robust routes to biocompatible, stable, isotonic high SAR suspensions for intravenous administration remain an active field of research, with significant technical challenges to be solved, including controlling the localized and the stray heat dose.

#### 1.1.2 Static‐Field Induced Forces

Exposure to static magnetic fields can align and lock the individual moments. The dependence of the average inter‐particle magnetic force on particle separation, *d*, changes from ≈*d* ^−6^ for randomly fluctuating to ≈*d* ^−3^ for locked moments^[^ ^14^ ^]^ which can reduce the inter‐particle interaction potential sufficiently to drive (oriented) aggregation. Field‐induced ordering of MNPs in suspension is well known, Kralj et al., showed how individual nanoparticles can be assembled into linear nanochains and nanobundles,^[^ ^24^ ^]^ a process that can be field reversible. Kralj et al. also show how reversible disaggregation following removal of the field can be prevented by sol–gel deposition of silica in‐field (a process which we categorize below as magnetic hydrogelation).

On the other hand exposure to magnetic field‐gradients (B∇B 10–500 T^2^ m^−1^) results in directional attractive forces that can produce magnetophoretic transport for dispersed nanoparticles^[^ ^16^ ^]^ or for submicron hydrogel‐stabilized MNP‐loaded particles,^[^ ^25^ ^]^ and; ii) internal deformations for macroscopic magnetic gels.^[^ ^26^ ^]^ Such approaches have been used for magnetophoretic capture of locoregionally‐^[^ ^27^ ^]^ and systemically‐administered MNPs in blood circulation.^[^ ^28^ ^]^ The latter may be limited by the trade‐off between the magnetic force and blood circulation time achievable, which usually have opposite dependencies on particle volume.

### 1.2 Magnetic Hydrogels/Inks Structure, Formation, and Classification

In this subsection we provide a classification of the types of magnetic hydrogels that can be formed by integration of MNPs and MNPCs (clusters or assemblies of MNPs). The nanocomposite hydrogels retain many of the native advantages of the gel component (high permeability, cell compatibility, printability, etc.) and they can also be manipulated in situ using applied AC‐ or DC‐magnetic fields. Weeber et al.^[^ ^29^ ^]^ reviewed the effects of polymer architecture in magnetic nanocomposite gels on performance for delivery applications, but none of these were structured materials.

**Figure 2:**
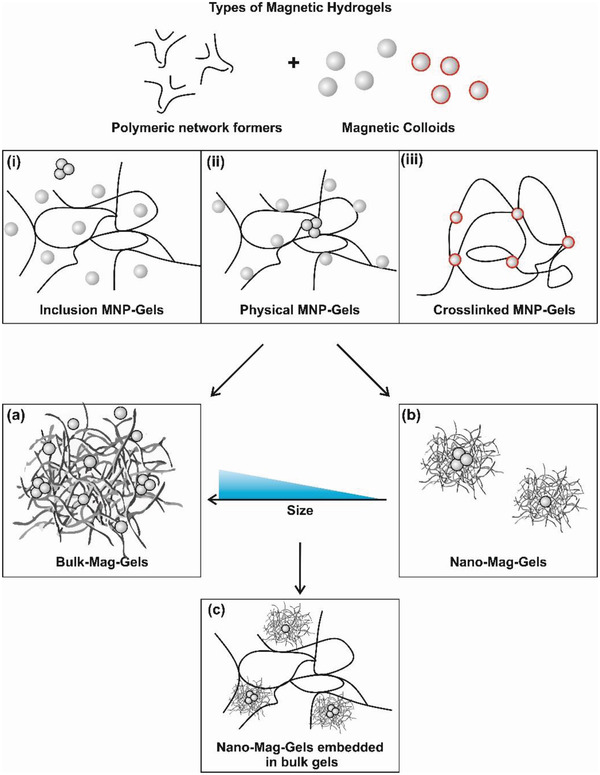
Schematic classification of different polymer gels incorporating MNPs. i) “Inclusion MNP‐Gels” with chemically cross‐linked networks and entrapped particles; ii) “Physical MNP‐Gels,” as above, with persistent physical interactions between MNPs and network‐forming chains; iii) “Crosslinked MNP‐Gels,” hybrid networks with particles cross‐linking chains by chemical or persistent physical interactions. Representations of; a) “Bulk‐Mag‐Gels,” and b) “Nano‐Mag‐Gels,” which can be formed from any of (i)–(iii). Finally; c) “Bulk‐Nano‐Mag‐Gels” in which Nano‐Mag‐Gels are incorporated in a bulk hydrogel network, these may also be formed by dispersing microscale Mag‐Gels. The formation of MNP clusters during nanocompositing may occur, hence the inclusion of some MNP trimers in (i)–(ii), (a)–(c). Clusters are less common in (iii) as single particles are used as cross‐linking points.

Simple embedding of nanoparticles within matrices can lead in principle to three distinct types of nanocomposites (**Figure** **2**), classified here by the types of interactions between the particles and network‐forming chains, and in turn by the type of network produced.^[^ ^4^, ^29^ ^]^ We suggest names for the magnetic versions of these different forms of nanocomposite suitable for this review. i) “Inclusion MNP‐Gels” in which particles are simply physically entrapped in the aqueous spaces between physically assembled or chemically or physically crosslinked hydrogels.^[^ ^30^ ^]^ Interactions of MNPs with the chains, if present, arise from van der Waals forces and hydrogen bonding. Such materials are intrinsically leaky. “Supramolecular MNP‐Gels” in which MNPs within micelles, or similar structures, interact weakly with the porous hydrogel network could also be included in this category; ii) “Physical MNP‐Gels” in which the physical interactions between the particles and network are stronger, reducing the average particle‐chain separation and extending the on‐chain residence times (free particles can be discounted),^[^ ^31^ ^]^ and; iii) “Crosslinked MNP‐Gels” in which particles crosslink separate chains or segments either through well‐defined chemical crosslinks or persistent strong physical interactions.^[^ ^32^ ^]^ The physical size of the composites formed, using any of the forms (i)–(iii), can vary enormously with limiting cases of; a) “Bulk‐Mag‐Gels,” dispersions of particles in a continuous macroscopic network, and; b) “Nano‐Mag‐Gels,” dispersions of MNP hydrogel nanocomposites in liquid. For completion Nano‐Mag‐Gels may be dispersed into bulk non‐magnetic hydrogels, to form; c) “Bulk‐Nano‐Mag‐Gels.” Ideally, in (b) and (c) the particles or nanogels are fully dispersed and homogeneously distributed throughout the material, although in many reports this is assumed rather than proven.

### 1.3 Magnetic Field Requirements for Controlled Structuring

**Figure 3:**
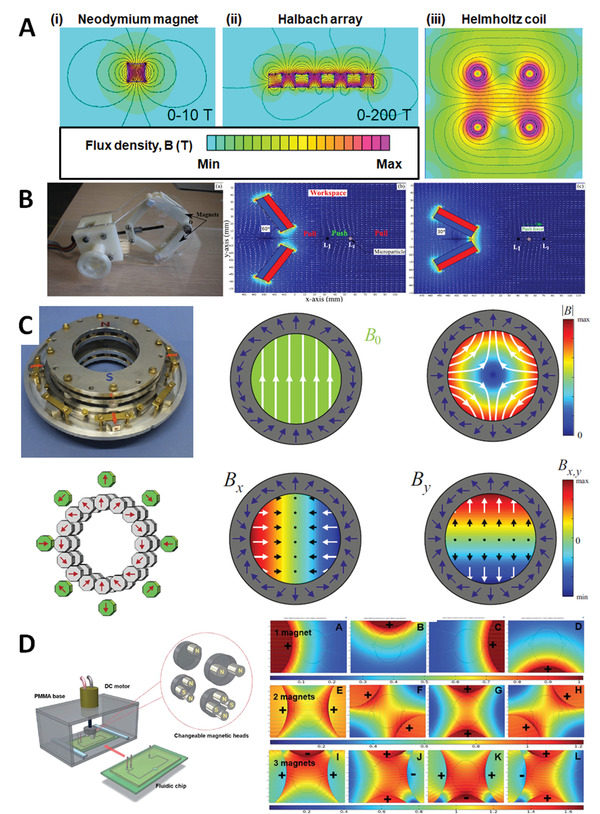
A) Schematic representation of commonly used magnet types with simulated of field lines for a neodymium alloy magnet a Halbach array and a Helmholz coil. B) Actuation systems consisting of two neodymium magnets attached to a moveable robotic arm. Reproduced with permission.^[^ ^34^ ^]^ Copyright 2018, Elsevier. C) Proof of principle arrangement of 16 (Halbach) paired permanent magnets (grey, producing a strong uniform dipolar field) surrounded by 8 magnets (green, producing a graded quadrupolar field), which can be rotated relative to each other, generating accessible uniform (several kilogauss) or dipolar fields, the B*_x_* and B*_y_* components of the flux are shown. Reproduced with permission.^[^ ^35^ ^]^ Copyright 2017, Elsevier. D) Schematic diagram of a fluidic chip inserted into a magnetic mixing system with easily exchangeable magnetic heads. Possible magnetic flux distributions of the magnet combinations are shown. Reproduced with permission.^[^ ^36^ ^]^ Copyright 2019, The Royal Society of Chemistry.

Successful implementation of magnetic fields during hydrogelation and additive manufacturing is critical to developing new functionalities. Generation of spatially‐controlled static magnetic fields and field gradients is a relatively mature technology applied across materials and biomaterials science, for instance in molecular particle imaging (MPI) and for guiding MNPs in the body. Yang and Zhang recently provided an extensive summary of the state‐of‐the‐art in the latter context.^[^ ^33^ ^]^ Permanent NdFeB alloy magnets (referred to here as “neodymium magnets”) typically generate field strengths (flux densities) at the surface of ≤0.65 T, **Figure** **3**A with strong spatial gradients that project a few cm into the sample providing alignment and translational forces (which are proportional to B∇B < 100 T^2^ m^−1^). Reasonably homogeneous static‐fields can be generated by appropriately oriented magnet pairs. On the other hand the gradient can be improved significantly, at least close to the face, by simple combinations, for example, Halbach arrays, Figure 3A. It has also been demonstrated that spatially‐defined push or pull forces can be generated through programmed robotic control of relative position and movement of pairs of (usually permanent) magnets, Figure 3B.^[^ ^34^ ^]^

More interestingly, user‐specified field, gradient direction and symmetry can be provided in printhead accessible spaces using single devices^[^ ^35^, ^36^ ^]^ that control the relative position and orientation of multiple permanent magnets, for example, Figure 3C,D. Electromagnetic coils can also be designed (e.g., as combinations of Helmholz and Maxwell coils) to provide homogeneous static‐fields, Figure 3A, into which samples can be placed. These approaches are usually limited to the 10's–100's range of mT, but they have the advantage that the homogeneous volume can be quite large. The magnitude, orientation and gradient can be controlled (in this case electrically), and again they can be designed to accommodate printheads and to move (so varying the static‐field over time). Despite usually being far from magnetic saturation (usually ≥1.5 T for MNPs) usable magnetic patterning can be demonstrated with routinely achievable static fields. This raises the possibility of improved responses with higher fields. High field, for example, NMR magnets which provide very high, very homogeneous fields over small volumes, could improve alignment for magnetic gelation of viscous formulations. MRI magnets have potential to provide (at some expense) the space needed for additive manufacturing approaches, but the printing equipment could not include magnetisable components. High field approaches to Mag‐Gel structuring are expensive and are not extensively reported.

Generation of spatially‐specified AC‐fields is also very interesting. Briefly, in most cases these fields are provided using a single resonant coil within a tuned circuit and the field is not directed. Clinical application of AC‐field hyperthermia has been beset by concerns including the measurement of stray heat dosage. Recent advances in MPI magnet technology,^[^ ^37^ ^]^ in combination with AC‐field hyperthermia could address spatial localization of AC‐field dose. MPI involves rastering a field‐free spot, within a strong static‐field, through the subject. The moments of particles entering the spot relax and that perturbation can be detected and hence the spatially‐specified MNP concentration can be determined. The approach also allows controlled heat dosage, as outside of the spot MNP moments are aligned and so have weak or negligible hyperthemic response. For applications in magnetic nanocomposite hydrogels the spatial localization of heat is more commonly provided by precise control of particle deposition provided, for instance, by advanced manufacturing of crosslinked Mag‐Gels, see below. However, MPI may provide new possibilities for in vivo dosage control from printed Mag‐Gel implants. Hence there is a toolbox of technologies emerging for development of magnetically oriented nanocomposites, and also or for magnetic additive manufacturing. Selected examples of these are described in more detail, in the context of the biomedical applications of Mag‐Gels, in Section 3.

### 1.4 Biological Compatibility Requirements of Mag‐Gels

The toxicity of MNPs has been found to strongly depend on dosage, surface chemistry, the cell/tissue type and the (in vivo) model used,^[^ ^14^ ^]^ but the oxide itself is reasonably tolerated and MNPs have been applied clinically. For hydrogels formed from a multitude of synthetic and bio‐derived polymers extensive studies over the past few decades^[^ ^38^ ^]^ have identified polymers and combinations of polymers that can be successfully used in biomedical and clinical applications.^[^ ^39^ ^]^ Fan et al. recently summarized the challenges for “bio‐inspired” hydrogels (without particles)^[^ ^40^ ^]^ confirming the view, noted above,^[^ ^8^ ^]^ that a major need is to mimic biological tissue by structuring bulk materials across multiple length scales.

In the case of Mag‐Gels the components are usually selected because of their demonstrated biocompatibility/minimal toxicity. There remains the possibility of toxicity emerging from nanocompositing of the components. It is more likely that issues could arise, particularly for Bulk‐Mag‐Gels, from their physical combination and/or from repeated application of magnetic stimulus. For instance, MNPs might leach from nanocomposite implants, or application of fields may result in enhanced permeability or the formation of voids. These issues must be addressed/eliminated on a case‐by‐case basis as approaches/materials mature toward application. In Section 3 many examples of Mag‐Gels will be described, and brief notes are provided on the reported biocompatibility. For more details on biocompatibility of nanoparticle loaded bio‐inks we refer readers to a recent review on this topic by Bakht et al.^[^ ^41^ ^]^

## 2 Magnetic Fabrication Technologies

In Section 2, Mag‐Gel fabrication techniques of increasing complexity (solidification, additive manufacturing, and advanced magnetic manufacturing), are reviewed with the emphasis on the technical aspects of producing nanocomposites with increasingly complex internal structuring. Applications follow in Section 3.

### 2.1 Magnetic Alignment During Solidification/Gelation—Magnetic Hydrogelation

**Figure 4:**
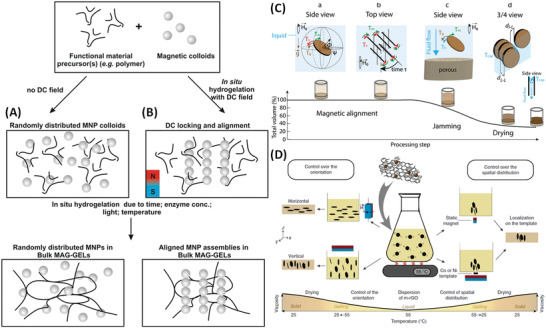
Magnetic hydrogelation. Schematic representations of hydrogelation process of magnetic colloids embedded in polymeric matrices. A) Hydrogelation can occur in the absence of a static‐field. B) Static‐field stimulus can be applied to induce alignment of magnetic colloids within the network, subsequent hydrogelation occurs over time and/or following an intervention, for example, enzymatically, UV crosslinking, or by temperature induced setting. C) Processing steps and torques involved during the magnetically assisted slip casting (MASC) process. Rotating magnetic fields were applied using a 300 mT neodymium magnet mounted on an electrical motor. Reproduced with permission.^[^ ^46^ ^]^ Copyright 2019, The Royal Society of Chemistry. D) Processing of gelatin‐based composites with magnetic control over the orientation or distribution of the m‐rGO flakes. Magnetic assembly is performed in the liquid phase with subsequent matrix consolidation to yield composites with tailored structures at both nano‐ and microscales. The wells were positioned on a neodymium magnet (250 mT). Reproduced under the terms of a Creative Commons Attribution 4.0 International License.^[^ ^42^ ^]^ Copyright 2016, The Authors, published by Springer Nature.

In the majority of the cases described below static‐fields are used to align particles during hydrogelation (or solidification) enabling formation of anisotropic patterns, an approach to fabrication/manufacturing that provides additional spatially‐specified functionality. Typical structures that can formed using magnetic hydrogelation are depicted in **Figure** **4**A,B. MNP alignment can also induce advantageous reorganization of diamagnetic components. Reported outcomes include one or several of the following: i) Formation of aligned percolating networks for directional electro‐conductivity;^[^ ^42^ ^]^ ii) mechanical hardening of the overall bulk material that depends strongly on the relative orientations of the MNP alignment with the shear and (when present) external magnetic field;^[^ ^43^ ^]^ iii) enhanced, orientation‐dependent hyperthermic responses, and;^[^ ^44^ ^]^ iv) topographically‐directed cue guidance for tissue engineering.^[^ ^45^ ^]^ Selected examples of these will be described in Sections 3.4–3.6.

It should be noted that magnetic hydrogelation can be induced by simple thermally‐activated processes including drying,^[^ ^46^ ^]^ or in situ time‐dependent chemical polymerization/crosslinking in the presence of a static‐field.^[^ ^42^ ^]^ For (bio)polymers that are available at scale hydrogelation induced on increasing temperature, of, for example, collagen,^[^ ^45^ ^]^ or on cooling, of, for example, gelatin,^[^ ^42^ ^]^ can provide opportunities to develop internal ordering in processes that have potential in manufacturing. MNP loading is key as it determines the strength of the magnetic response but increasing content can force MNP aggregation which degrades any functionality. In situ photopolymerization can also be used at scale, but in this case the MNP content trade‐off is further complicated by the need for sufficient light transmission, given the efficiency of the selected photochemistry.^[^ ^7^ ^]^

Alignment during magnetic hydrogelation arises from locking of MNP moments by a static field, most commonly by applying neodymium magnets to the viscous formulations. As noted above, this alters the distance dependence of the average inter‐particle dipolar magnetic interaction,^[^ ^47^ ^]^ with the resulting increased dipolar attraction driving chain formation which can accelerate (depending on concentration) as the chains grow. The magnetic ordering of the final composite is usually retained following gelation on removal of the field. AC‐fields have also recently been shown to induce formation of aligned chains in suspension.^[^ ^18^ ^]^ Hence there may be possibilities for AC‐field induced alignment during hydrogelation, at least during the early stages. It was recently demonstrated that chain formation and alignment can arise in magnetic nanocomposite inks during processing without the use of an external field. In this case 23 nm nanocubes, which have stronger magnetocrystalline anisotropy than spheres of equivalent volume, were found to align within electrospun fibres.^[^ ^48^ ^]^

In all cases sedimentation and the potential (when a single magnet is used) of the spatially decreasing static‐field, that is, the gradient, to exert translational forces across the sample may result in lack of homogeneity in the final Mag‐Gel. For 3D applications gel homogeneity should be confirmed rather than inferred. For 2D culture formats, this can be less of an issue. Cells are usually grown on the upper side, that is, the surface for which the alignment/topography is generated and observed, for example, by optical microscopy; so the ordering in the sub‐structure may be less important. Regions of different alignment may also be specified across the sample volume, for example, through control over the static‐field strength, orientation, or rotation. The persistence length/spatial averaging of encoded magnetic enhancements reflect the size of the aligned regions. The examples described in Section 3 also show how organization on multiple length scales can be a strength, providing an additional level of control over magnetic response through the manufacturing process.

### 2.2 3D Printing for Structuring Nanocomposite Materials—Additive Manufacturing of Magnetic Materials

**Figure 5:**
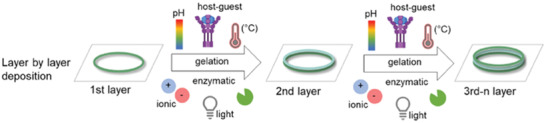
Bottom‐up approach with layer‐by‐layer deposition of a bio‐ink to produce 3D scaffolds, including gelation (e.g., light, ionic, pH, temperature, host–guest interaction, enzymatic) between depositions of single layers. Reproduced with permission.^[^ ^50^ ^]^ Copyright 2020, American Chemical Society.

Over the past two decades, motivated in part by the potential applications of patterned 3D structures with hierarchical‐organization,^[^ ^8^, ^49^ ^]^ 3D printing technologies have evolved rapidly. Additive manufacturing technologies, in particular, have underpinned development of spatially‐specified biofabrication for rapidly prototyping tissue disease models, which increasingly recapitulate native organization of biochemical and mechanical properties. Spatiotemporally controlled delivery of biomechanical and chemical cues from the nanocomposites for tissue engineering applications remains a challenge,^[^ ^38^ ^]^ but these developments may in time contribute to reproducible organ printing on small scale.^[^ ^50^ ^]^ Recently Schwab et al. summarized how additive manufacturing technologies, especially extrusion‐based and stereolithographic approaches, can be used for automated fabrication of hierarchically organized constructs and how rheological factors affect printability/determine shape fidelity.^[^ ^50^ ^]^ These two approaches, in particular, provide great scope for sequential printing with the same or different formulations, and perhaps with controlled extent of crosslinking and/or inclusion of different (bio)functionalities to build up complex stable layered structures with good spatial control, as represented schematically in **Figure** **5**. To date the reports have predominantly been for non‐magnetic formulations.

For example, Schwab and co‐workers fabricated hierarchical scaffolds made from tyramine functionalized hyaluronic acid and type 1 collagen, with fine control over the position and alignment of fibres enabled by extrusion printing and enzymatic crosslinking.^[^ ^51^ ^]^ Despite the demonstrated flexibility for fabricating spatially‐patterned structures of different feature size (using multiple extrusion nozzles^[^ ^52^ ^]^), and the wide choice of synthetic and bio‐derived printable materials, generating organization within a single object over lengths spanning from the nano‐ into the macro‐scale remains a huge challenge.

In general the same requirements (homogeneity of the samples, rheological shear‐thinning) apply when magnetic inks are used in otherwise conventional additive manufacturing. The use of printed magnetic nanocomposite formulations, can increase architectural complexity and provide temporally‐controlled stimulus in spatially‐specified release patterns, mechanical deformations, or permeability changes.^[^ ^53^ ^]^ In the following sections we will describe recent progress toward these goals in the context of magnetically responsive hydrogels.

### 2.3 Approaches to Advanced Magnetic Additive Manufacturing

**Figure 6:**
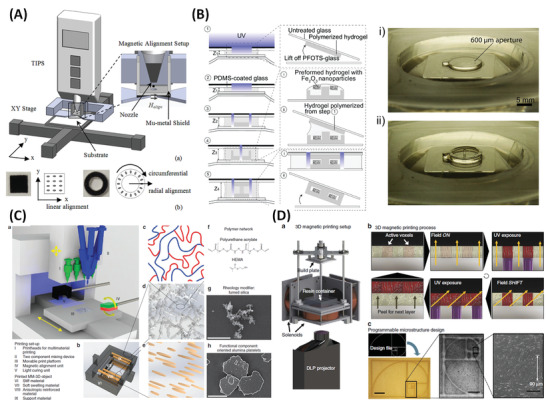
A) Magnetic inkjet printing. a) Schematic of an inkjet printing and magnetic alignment setup. b) Photos and schematics of an x‐aligned square, and a radially‐aligned ring sample. Reproduced with permission.^[^ ^54^ ^]^ Copyright 2014, AIP Publishing LLC. B) Magnetic layer by layer deposition. Schematic of step‐by‐step device fabrication within a PDMS chamber. Reproduced with permission.^[^ ^55^ ^]^ Copyright 2018, MyJoVE Corporation. C) Magnetic extrusion‐based printing. Schematics of the MM‐3D platform for printing heterogeneous composites. a) Direct ink‐writing hardware equipped with multiple I) dispensers, II) mixing unit, III) movable head and table, IV) magnet, and V) a curing unit. b) Example of a print designed to change shape on stimulus by incorporation of inks of different stiffness and swelling. c–e) Illustrations of typical ink constituents. c) Oligomers and monomers that form the base material and are crosslinked after deposition to generate a polymer network. d) Fumed silica (FS) nanoparticles that percolate throughout the resin. e) Anisotropic particles oriented by the external magnetic field. f) Chemical structures of typical ink monomers/oligomers. g) SEM image of FS particles. Scale bar, 500 nm. h) SEM image of alumina platelets. Scale bar, 10 mm. Reproduced under the terms of a Creative Commons Attribution 4.0 International License.^[^ ^56^ ^]^ Copyright 2015, The Authors, published by Springer Nature. D) Magnetic stereolithography. a) This 3D system uses DLP to UV‐photopolymerise in a movable static‐field generated using solenoids, which, b) aligns and selectively polymerizes groupings of voxels programmed to have specific reinforcement orientation within each layer. The build plate peels after a layer is complete to print additional layers. c) Example of a reinforced micro‐architecture (a gold rectangle with feature sizes ≤90 mm. Scale bars 2, 500, and 50 mm (left to right). Reproduced under the terms of a Creative Commons Attribution 4.0 International License.^[^ ^57^ ^]^ Copyright 2015, The Authors, published by Springer Nature.

Conventional 3D printing setups/methodologies can be re‐engineered and modified to include permanent (stationary or rotating) magnets, and thus add an additional dimension of internal hierarchical organization to the printed magneto‐responsive materials. This approach can be considered a sub‐category of what is often termed “4D printing,” where an additional element of control, or degree of freedom, is exploited, for example, by application of external fields.^[^ ^9^ ^]^ Here four distinct types of magnetic additive manufacturing will be described which broadly follow the conventional additive manufacturing categorization, in which a conventional method was modified by inclusion of permanent magnets and use of magnetic inks: i) Magnetic inkjet printing; ii) magnetic layer‐by‐layer deposition; iii) magnetic extrusion‐based printing, and; iv) magnetic stereolithography (**Figure** **6**). For each method, we briefly describe the implementation of the magnetic field and how this contributes to internal structuring and hence to the capabilities of the printed structures.

#### 2.3.1 Magnetic Inkjet Printing

Song et al. presented the first case of i) magnetic inkjet printing where the deposited materials were aligned in the stream by including an electromagnet below the nozzle (Figure 13A).^[^ ^54^ ^]^ The nozzle (60 µm orifice) was shielded to prevent field induced aggregation of the 30 vol% MNP suspension. The controller and the alignment field direction were fixed during printing, whereas the substrate paper/stage was moved either by a stepper motor with 50 µm resolution, or rotationally. Orientation‐dependent permeability was confirmed for square‐ or ring‐shaped thin films (Figure 6A) with axial or radial magnetic patterns achieved by translating or rotating the stage at 2 Hz during ink‐jetting, respectively, at fixed field strength of 10 mT. This was the first demonstration of fine control over both macrostructure, as well as, internal ordering in one manufacturing step, which was not achievable by the previously described magnetic manufacturing technologies. 3D printing with magnetic hydrogelation either pre‐ or post‐ deposition (i.e., in the barrel, or on the substrate, respectively) could provide similar structures. However that would require multiple processing steps that may be difficult to implement reproducibility.

#### 2.3.2 Magnetic Layer‐by‐Layer Deposition

Chin et al. described a ii) magnetic layer‐by‐layer deposition technique, whereby each layer (although in this case it is not provided by a printing approach) is photo‐cured and bonded to the next layer forming complex 3D structures (Figure 6B).^[^ ^55^, ^58^ ^]^ In these examples, magnetic inks were used to generate micro‐machines with permanent magnetization. External neodymium disc magnets were used to generate movement/action including a “locking mechanism” with precise actuation. This magnetic layer‐by‐layer technology is an interface between conventional 3D extrusion printing and digital light processing (DLP) technologies. Again, control over both macrostructure, as well as the internal ordering of particles can be achieved by control over fabrication, inclusion of the static fields during processing, and by the type of Mag‐Gel network.

#### 2.3.3 Magnetic Extrusion‐Based Printing

Kokkinis et al. described another type of magnetic additive manufacturing; (iii) magnetic extrusion‐based printing.^[^ ^56^ ^]^ Although extrusion‐based printing is a layer‐by‐layer method, we differentiate it here from the previous example due to the differences in the experimental setup and the use of nozzles. In this first mode a neodymium magnet was positioned in front of the moving extrusion nozzle to control internal alignment of magnetized stiff alumina platelets in a polyurethane acrylate‐based ink (Figure 6C).^[^ ^56^ ^]^ By controlling the rotation of the neodymium magnet (40 mT at 8.3 Hz) during printing, magnetized alumina platelets were formed into complex internal architectures, with controlled pitch. The simplest case, a helical millimeter‐scale staircase, was demonstrated.^[^ ^56^ ^]^ The rotation frequency used, 8.3 Hz, provided sufficiently high drag forces on the platelets to promote their biaxial alignment within the plane of the rotating field, and masking and UV‐curing after printing each layer ensured additional control over spatial localization. It is also interesting that radial platelet concentration gradients (from 1.85 to 0.37 vol%), from the center toward the edge, could be achieved by mixing (at varying vol%) a platelet‐loaded stem suspension with a pure resin ink in a dual‐component dispenser.

Furthermore, by selecting platelet orientation in the printing (xy) plane, nanocomposites could be prepared with increased strength (by 49%) and elastic modulus (by 52%) in the tensile loading direction, when the orientation was in‐plane (perpendicular to z), as compared to out‐of‐plane (parallel to z).^[^ ^56^ ^]^ This was also accompanied by a 30% increase in the swelling strain for the in‐plane orientation. The researchers automated this printing procedure allowing (consecutive and cyclic) movement of the sample between three stages for each layer of: i) 3D printing; ii) alignment with the rotating magnet, and; iii) crosslinking (at a blue‐diode station). Clearly, magnetic extrusion‐based printing is a versatile tool for generating nanocomposite structures that resemble complex hierarchical designs, or that are spatially reinforced. This fabrication technique is particularly useful for generating millimeter‐scale objects with nanometer‐scale structuring embedded, hence it could enable unparalleled replication of biological designs in synthetic materials.

Another option is to place static‐fields (neodymium or electromagnets) directly around the extrusion‐nozzle. This second mode was demonstrated for programming alignment of ferromagnetic NdFeB microparticles dispersed in elastomer matrices,^[^ ^59^ ^]^ with application of the field shown to impart patterned magnetic polarity to 840 µm (nozzle diameter) printed filaments. In this way, innovative structures could be printed of auxetic metamaterials (increasing perpendicular cross section, and thus actuation on application of perpendicular static‐fields).^[^ ^59^, ^60^ ^]^ Kim and colleagues demonstrated encoding of ferromagnetic domains in a printed filament with an alternating magnetization pattern, by switching the applied field direction in situ during extrusion.^[^ ^59^ ^]^ On application of a uniform 200 mT static‐field the straight millimeter‐scale printed struts transformed readily into an M shape within 0.1 s and were reported to revert back to the original shape within 0.2 s on removal of the field. As the field was homogeneous the effect apparently arises from alignment‐induced internal forces. This innovative magnetic extrusion‐based printing method could be extended to multiple hydrogel‐based nanocomposite inks, providing soft, (relatively) fast‐responding, shape‐shifting, and electronically‐actuated devices for biomedical applications.

#### 2.3.4 Magnetic Stereolithography

Most conventional extrusion‐based printing techniques have stereolithographic equivalents, this is also the case for printing magnetic nanocomposite inks. In iv) magnetic stereolithography (Figure 6D) millimeter‐scale objects can again be formed from components that embed nanometer‐scale spatial organization. Martin et al. used this approach to create highly programmable discontinuous fibres inspired by the biologically relevant structures of abalone shells, peacock mantis shrimp and mammalian cortical bone.^[^ ^57^ ^]^ The authors used DLP to UV photo‐polymerize resin and modified the frame of the instrument to allow simultaneous application of static‐magnetic fields, generated using either a pair of computer controlled solenoids or a rotating neodymium magnet (Figure 6A). Once again to build the internal architecture, magnetized alumina particles were used, dispersed in this case in photo‐curable aliphatic urethane diacrylate and isobornyl acrylate (1:3 ratio by weight). Good structural resolution was achieved, with features as small as 90 µm (Figure 6D‐c) and an upper size limit for the constructs of ≈10 × 10 × 10 cm. The method allows formation of materials with superior spatial control of the exact orientation of the magnetized particles, similar to the organization of, for example, osteon microstructures with concentric reinforcement orientation and “monolithic” parts. The authors demonstrated this concept by changing the angle of the incorporated alumina platelets, from 0 to 90°, in 30° steps, with progressively decreasing relative tensile strength measured with increasing angle.^[^ ^57^ ^]^ Fine control over architecture can be used to program orientations of components around defects, for example to reinforce the structural and mechanical integrity. Hence magnetic manufacturing methods could in principle be used to generate structures with inbuilt crack‐propagation direction combined with self‐healing capability, providing possibilities for generating temporary interfaces/fractures.

Safaee and Chen prepared resin‐MNP nanocomposites (using isobornyl acrylate as a viscosity modifier) with ultra‐high spatial resolution by a magnetic stereolithography approach in which a conventional polymerization setup was equipped with a linear array of neodymium magnets with vertical steps in the magnet holder (to generate a field‐ and hence an MNP‐gradient), within a set of Helmholtz coils (to generate a uniform static‐field, which produces gradient‐free MNP alignment in the resin in the absence of the array).^[^ ^61^ ^]^ MNP gradients (from 0 to 4 wt%) were achieved, typically with Young's modulus changing from 0.8 to 1.5 GPa along the gradient. The study, enabling preparation of materials with more predictable properties, establishes a framework for design and manufacture of functionally graded polymer nanocomposites. It also identified the possibility of applying more complex gradients, or preparing layered systems of differing texture, which would have applications across many functional biomaterials.

In **Table** **1** a summary of the magnetic fabrication techniques described in Sections 2.1–2.3 is provided, including the technological and ink/material requirements of each method, and noting their general advantages and disadvantages. We also note the types of networks, as described in Section 1.2, that can be used as precursor inks for each fabrication method. The review to this point identifies the most recently reported magnetic fabrication methods, including magnetic extrusion‐based printing and magnetic stereolithography which in principle allow i) fabrication of complex structures of different size with internal‐ordering over multiple length scales; ii) programming of spatiotemporally‐specified magnetic responses into the fabricated constructs, that is, time‐dependent responses under different stimuli with spatial‐specification.

**Table 1:** Summary of the magnetic additive manufacturing processes, with highlighted modes of action, advantages and disadvantages, and details of the magnetic fields used

Magnetic fabrication technology and main capabilities	Technological requirements	Advantages	Disadvantages	Inks/materials requirements	Relevant references
Magnetic hydrogelation Can program structural features and orientation dependent responses.	Simple homogeneous static fields are required (0.1–1 T).	Facile method Scalable Allows retention of biological properties of inks.	Uniaxial orientation of chains only. Difficult to fully align MNPs with easily achievable fields and to generate complex patterns. Responses imprinted across entire Mag‐Gel.	Inks must allow setting, either by: thermogelation (e.g., gelation) drying (jamming) or photopolymerization to lock‐in MNPs and generate stable structures with high fidelity. Can be used to form all types of networks (Inclusion, physical, and crosslinked MNPs‐Gels).	[42, 45, 46, 62]
Conventional (extrusion‐based and digital light processing) 3D printing of magnetic materials. Can program structure and hence generate spatially‐dependent responses.	Does not use any magnetic fields during fabrication.	Uses established methodologies for magnetic inks. Easy to use and well‐established.	Structures may still require post‐printing stabilization (similarly to conventional printing). Still cannot fabricate more complex structures (e.g., orientation within a given layer).	Inks must be printable, that is, MNPs cannot drastically change rheology and must allow for light polymerization using digital light processing. Are only suitable for inclusion and physical MNP‐Gels. Digital light processing techniques can generate networks of widely variable crosslinking.	[7, 53, 63]
Magnetic inkjet printing Can program intrinsically complex structures across all length scales.	Inclusion of electromagnets or permanent magnets below/around nozzle.	Allows complex field‐induced patterning of included MNPs.	Limited to low viscosity inks.	As a result is effectively limited to inclusion and physical MNP‐Gels.	[54]
Magnetic layer‐by‐layer deposition Can program intrinsically complex structures across all length scales.	External neodymium disc magnets or electromagnets are included in setup.	Can be used to generate movement/action including “locking mechanisms” with precise actuation.	Interfacing technology requiring more time and setup input.	Magnetic inks must allow curing either by thermogelation, light polymerization or other methods. Can typically only be used with inclusion and physical MNP‐Gels.	[55, 58]
Magnetic extrusion‐based printing Can program intrinsically complex structures across all length scales with spatially‐specified magnetization, so providing enhanced temporal magnetic responses enhanced responsivity.	Permanent magnets (also can be rotated) can be attached in close vicinity to the nozzle.	Allows structuring across all length scales or programming of responsiveness (magnetization) into printed filaments.	Inclusion of MNPs cannot significantly change the ink rheology/printability. Interfacing technology requiring more time and setup input.	Inks must be printable, that is, MNPs cannot drastically change rheology. Extrusion‐based techniques can typically only be used with inclusion and physical MNP‐Gels.	[56, 59, 60]
Magnetic stereolithography Can program intrinsically complex structures across all length scales with spatially‐specified magnetization (with higher printing resolution possible), so providing enhanced temporal magnetic responses enhanced responsivity.	Simultaneous application of static‐magnetic fields using either a pair of computer controlled solenoids or a rotating neodymium magnet is achievable in a custom‐ designed frames around printing baths.	Allows structuring across all length scales or programming of responsiveness (magnetization) into printed filaments (with higher printing resolution).	Interfacing technology requiring more time and setup input.	Inks must be printable, that is, MNPs cannot drastically change rheology and must allow for light polymerization. Digital light processing techniques in principle can generate all types of networks.	[57, 64]

## 3 Stimulus Response of Magnetic Hydrogels and their Biomedical Applications

In this section the types of biologically relevant responses and the Mag‐Gel structures used are summarized, both those generated conventionally providing the benchmark, as well as those generated using magnetic fabrication technologies. The examples are related back to the classification of advanced magnetic additive manufacturing techniques in Sections 2.3.1–2.3.4.

### 3.1 AC‐Field Mediated Hyperthermic Release

**Figure 7:**
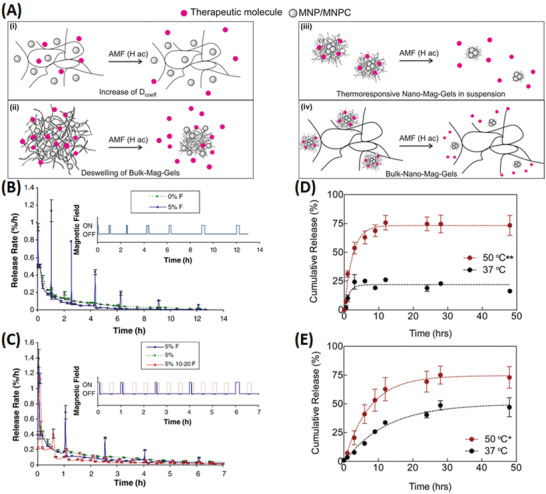
A) Scheme showing typical strategies for AC‐field induced Mag‐Gel therapeutic delivery, including; i) temperature‐induced increase in molecular diffusion, and deswelling of; ii) Bulk‐Mag‐Gels, or; iii) Nano‐Mag‐Gels composed of thermoresponsive components, and; iv) inclusion of thermoresponsive Nano‐Mag‐Gels in non‐response bulk matrices. B) Vitamin B12 release from Bulk‐Mag‐Gels on application of pulsed AC‐field (pulses are labelled “F”), % represents vitamin B12 loading by weight in pNIPAM–TEGDMA (Triethylene glycol dimethacrylate) nanocomposite. C) Methylene blue release under the same conditions. Reproduced with permission.^[^ ^66^ ^]^ Copyright 2008, Elsevier. D) Release kinetics of doxorubicin from pNIPAM‐co‐Am Nano‐Mag‐Gels was observed to be significantly greater (**p < 0.01) when exposed to temperatures above the volume phase transition temperature (VPTT). E) When Dox loaded Nano‐Mag‐Gels were encapsulated in the GelMA matrix, release was also significantly greater (*p < 0.05) when exposed to *T* > VPTT. Reproduced with permission.^[^ ^67^ ^]^ Copyright 2017, The Royal Society of Chemistry.

The field of nanomaterials for AC‐field hyperthermia is well developed, so we will not attempt to provide an exhaustive listing but have selected some recent highlights that illustrate the different possibilities. Commonly used polymers include gelatin, alginate and thermally responsive poly(*N*‐isopropylacrylamide) and poly(*N*‐isopropylacrylamide‐*co*‐acrylamide) and there are reports of Nano‐, Micro‐, and Bulk‐Mag‐Gels.^[^ ^65^ ^]^ Diffusive and deswelling contributions have been noted for AC‐field responsive release from Bulk‐Mag‐Gels. In (**Figure** **7**A–C, left) pioneering work from the Hilt Group is shown, where MNPs in thermoresponsive pNIPAM cast as discs,^[^ ^66^ ^]^ show continuous (diffusive) and pulsatile (AC‐field responsive deswelling) release of molecules, including vitamin B12 (Figure 7B) and methylene blue (Figure 7C).^[^ ^66^ ^]^ The same group showed similar controlled drug release using pNIPAM–PEGDMA (PEG dimethacrylate) formulations.^[^ ^66^ ^]^

Potential advantages of Bulk‐Nano‐Mag‐Gels (Figure 7E,F, right) arise due to co‐locating the MNPs, therapeutics and thermally‐responsive part of the network both to amplify the thermal response and reduce passive (AC‐field‐off) leakage. In a recent study, Jalili et al. fabricated Bulk‐Nano‐Mag‐Gels from pNIPAM‐*co*‐Am for doxorubicin (Dox) delivery (Figure 7D), the materials showed an interesting increase in the lower critical solubility temperature (LCST) toward more biologically relevant temperatures.^[^ ^67^ ^]^ The authors demonstrated how thermally responsive release from the Nano‐Mag‐Gel component (Figure 7D) is not compromised by encapsulation in a photo‐crosslinkable gelatin methacrylate (GelMA) network (Figure 7E). Although printing was not described, injectability tests using conventional surgical needles suggest sufficient shear‐thinning for printability. In most reports gelation suppresses the hyperthermic response significantly, largely due to loss of Brownian contributions to heating but also in many cases because of induced aggregation.^[^ ^68^ ^]^ However it is usually possible to produce sufficient temperature increases at reasonable concentrations for many biomedical driven applications, including in vivo tumor eradication or controlled release platforms.^[^ ^23^, ^69^ ^]^ In Section 3.7 we also describe an example of extrusion 3D‐printed Mag‐Gels for in vivo combined hyperthermic tumor eradication and tissue repair.

### 3.2 Directional Hyperthermic Responses

Hu et al. used magnetic fabrication during in situ hydrogelation of polyacrylamide hydrogels.^[^ ^44^ ^]^ Details of the magnetic field used were scant, but by controlling the field exposure time different extents of MNP alignment were achieved, from isotropic, to short chains, to extended parallel single particle chains persisting throughout the gel, and up to chains comprised of multiple particles. For aligned Mag‐Gels the bulk elastic compression modulus was 1.27‐fold higher when the force was applied parallel (1.9 × 10^4^ Pa) as opposed to perpendicular (1.5 × 10^4^ Pa) to the direction of alignment. While the hyperthermic responses (both initial slopes, *dT*/*dt*_0_, and plateaus, Δ*T*_max_) were increased, as compared to non‐aligned materials, by factors of up to 6–8 and 1.5–2, again with the field direction parallel and perpendicular, respectively. These advantages were used to demonstrate stimulated (thermogenic) release of Dox at a rate that could be manipulated through the orientation of the AC‐field with respect to the alignment axis.

In our previous work in this field, we demonstrated 3D printable responsive magnetic hydrogels composed of magnetic iron‐oxide nanoflowers and Pluronic polymers that upon AC‐field irradiation (642 kHz, 16 mT) provided localized spatiotemporally‐specified heating.^[^ ^69^ ^]^ The temperature jumps achieved were sufficient to increase the diffusion of methylene blue, which was released from the patterned grids.^[^ ^69^ ^]^ More recently we further demonstrated that the heating capabilities of such grids can be enhanced by modulation of inter‐MNP interactions (preventing formulation‐induced aggregation which suppresses hyperthermia) by addition of graphene oxide flakes as spacers.^[^ ^23^ ^]^ The improved responses provide possibilities for patterned and directional hyperthermic release. In time these approaches may be incorporated into automated magnetically‐controlled systems that provide timed and spatially localized release, for example, in multi‐phenotypic differentiation of cells (e.g., for controlled organoid growth), or in patterned hyperthermia‐induced release (e.g., to spatially catalyze biological reactions via the Fenton reaction^[^ ^63^ ^]^).

### 3.3 AC‐ and Static‐Field Induced Structural Deformation

**Figure 8:**
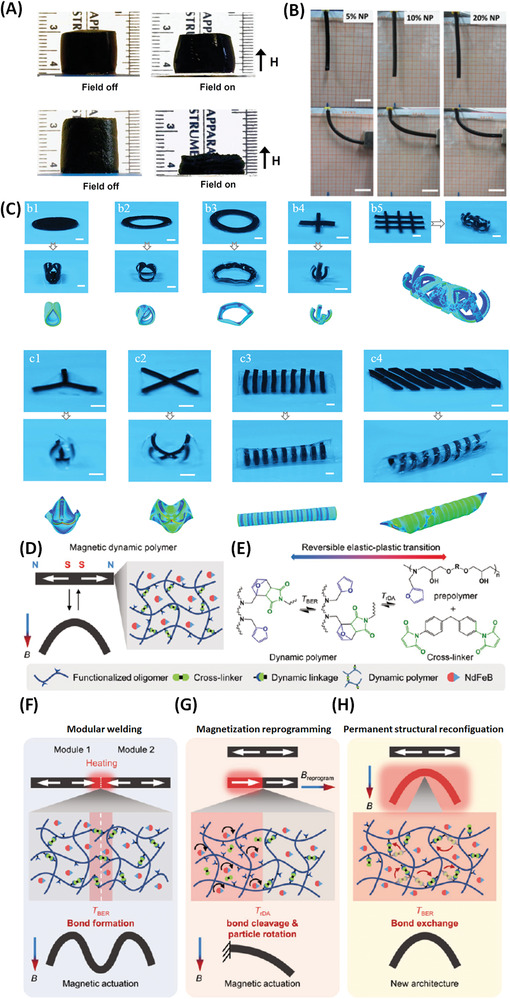
Structural deformation responses in Bulk‐Mag‐Gels. A) Simple contraction/extension and strain induced by static‐fields. Stress versus strain curves for nano‐ and macroporous Bulk‐Mag‐Gels subjected to compression. A cylinder of a nanoporous ferrogel (13 wt% MNPs) reduced in height by ≈5% (upper pair of images) when subjected to a vertical field‐gradient of ≈38 A m^−2^ generated by a bar magnet placed underneath. Under the same conditions a macroporous ferrogel of similar loading deformed by ≈70% (lower pair). Reproduced with permission.^[^ ^70^ ^]^ Copyright 2011, National Academy of Sciences. B) Static‐field induced (by a permanent NdFeB alloy magnet) bending in alginate/polyacrylamide with varying MNP wt% on application of a field‐gradient. Scale bars: 2 cm. Reproduced with permission.^[^ ^71^ ^]^ Copyright 2015, The Royal Society of Chemistry. C) AC‐field (17.8 kA m^−1^, 203 kHz) induced shape‐morphing (quasi‐2‐ and 3‐D) hybrids of MNP/pNIPAM strips patterned onto elastomer. Representative hybrids are shown with matching Mag‐Gel and elastomer shapes. Illustrative finite element analysis representations are included. Scale bars 5 mm. Reproduced with permission.^[^ ^72^ ^]^ Copyright 2019, American Chemical Society. Schematics of the mechanism and functions of the “magnetic dynamic polymers” (MDP). Schematics representations of; D) MDP composition, NdFeB microparticles are embedded in a dynamic polymer bearing reversible chemical bond; E) Reversible elastic–plastic transition (due to network topology transitions at different temperatures); F) welding of modules at *T*–*T*_BER_; G) Magnetization reprogramming by bond cleavage and particle rotation under a magnetic field at *T*_rDA_; (H) Magnetically‐guided permanent MDP plastic reconfiguration by stress relaxation at *T*_BER_. Reproduced with permission.^[^ ^73^ ^]^ Copyright 2021, Wiley‐VCH GmbH.

For Mag‐Gels of different sizes static‐fields can provide reversible contraction/extension (**Figure** **8**A),^[^ ^32^, ^70^ ^]^ which is tuneable through pore size‐dependent reduction in rigidity and increase in hydraulic conductivity, and even bending (Figure 8B) responses.^[^ ^71^ ^]^ Such responsive Bulk‐Mag‐Gels can be incorporated into more advanced designs including high‐throughput 3D cell culture formats. Recently microscale magnetically‐actuated cell‐laden hydrogels, formed into ≈2 cm chip‐like arrays, with individual actuators of the order of ≈1–2 mm, were used to assess the utility of mechanically responsive microenvironments for directing cellular outcomes. Oscillation with up to 60% strain effected mechano‐transduction in cultured cells, revealing cellular strain‐thresholds and saturation behaviors unlike those previously shown for non‐dynamic 2D and 3D culture systems.^[^ ^26^ ^]^ In this case actuation was achieved using the field generated by a permanent NdFeB alloy magnet (0.53 T at the surface) controlled by a displacement stage.^[^ ^26^ ^]^

AC‐field responsive (hyperthermically driven) volumetric changes in magneto‐thermally responsive hydrogels, typically MNP/pNIPAM Bulk‐Mag‐Gels which, as noted above, can be used for stimulated release, can also drive controlled shape change, or can induce self‐healing by rapid temperature driven bond reformation. For example, volumetric change can be achieved in magnetic pNIPAM hydrogels placed in microfluidic devices to remotely control flow, providing smart, AC‐field responsive, valves.^[^ ^74^ ^]^ Hydrogels such as poly(*N*‐isopropylacrylamide‐*co*‐acrylic acid) (pNIPAM‐AAc) can be photo‐patterned to create a wide range of actuatable and self‐folding microstructures.^[^ ^75^ ^]^ Indeed, reversible mechanical “soft” robotic motion can be achieved with this cross‐linked material due its strong thermal‐ and pH‐dependent responses. By patterning hydrogels of these types with stiffer non‐swellable elements, controllable functions such as micro‐gripping can be encoded arising from the differences in expansion on stimulus.

Mechanical responses could in principle be remotely controlled by embedded MNPs inside the porous pNIPAM‐AAc layer. By patterning a similar formulation of Bulk‐Mag‐Gel strips onto elastomer substrates (Figure 8C), it was shown^[^ ^72^ ^]^ that finely controlled complex shape changes can be induced without direct contact by AC‐field stimulus (generated by a commercial induction‐heating system, 17.8 kA m^−1^, 203 kHz) on reaching the LCST of the thermoresponsive component. This was observed for different formulations, and hence different rates of temperature increase, indicating that an on/off response was elicited once the system reached the threshold. The authors also noted that these materials could in principle be printed to form increasingly complex shapes and they demonstrated combined shape change and field‐gradient induced transport. Other reports demonstrated the use of AC‐driven hyperthermia to increase the time needed to re‐form host–guest bonds and so self‐heal severed parts of a Bulk‐Mag‐Gel,^[^ ^76^ ^]^ and programme reconfigurable regions within similar constructs which show either magnetic self‐healing or actuation by using a pair of (in‐house built) electromagnetic coils providing a homogeneous static‐field of up to 0.1 T (Figure 8D–H).^[^ ^73^ ^]^ Kuang et al. described “magnetically dynamic polymers” which contain thermally sensitive bonds, which as a result can be spatially‐directed by application of an AC‐field (Figure 8D,E).^[^ ^73^ ^]^ This capability, arising from non‐contact control over chemical change, enables three distinct physical modifications: i) Modular welding, that is, self‐healing or magnetically driven assembly (Figure 8F); ii) magnetization reprogramming, and thus the possibility of selected direction of movement, for example, field‐gradient specified transport (Figure 8G), and; iii) permanent structural reconfiguration, allowing in situ reformation of selected structural forms (Figure 8H). Through combination of these responses, that is, sequential application of AC‐ and DC‐ stimulus, new possibilities emerge for remote navigation through tight junctions / tortuous environments. Field‐gradients, typically achieved using permanent neodymium alloy magnets, have also been applied for more “conventional” guiding of Bulk‐Mag‐Gel scaffolds, for example in one case static fields (0.38–0.48 T) were used for positioning regenerative implants^[^ ^77^ ^]^ and anticancer materials at tumor sites.^[^ ^78^ ^]^

**Figure 9:**
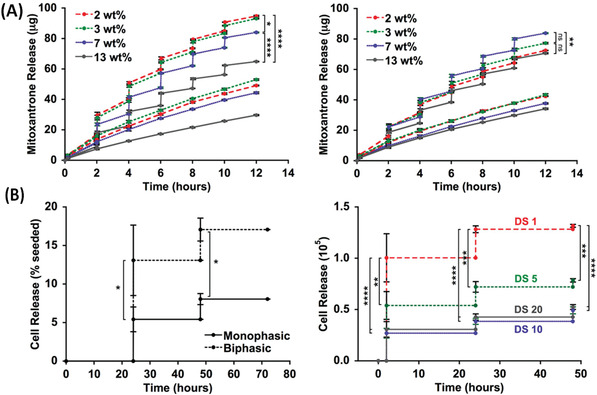
A) Mitoxantrone release from the biphasic (left) and monophasic (right) Bulk‐Mag‐Gels with different MNP loading, 2, 3, 7, and 13 wt%, as depicted in the legend insert in graphs, following no stimulation (bottom curve) or magnetic field stimulation for 2 min at 1 Hz every 2 h. All ferrogels were initially loaded with 150 µg mitoxantrone. B) Release of viable cells from; left monophasic and biphasic Bulk‐Mag‐Gels of the same MNP concentration following magnetic field stimulation for 2 min at 1 Hz every 24 h; right biphasic peptide gels of varying RGD density cells under the same conditions. Reproduced with permission.^[^ ^79^ ^]^ Copyright 2014, Wiley‐VCH GmbH.

Induced volumetric changes in Bulk‐Mag‐Gels, such as the example from the Mooney Group, open up possibilities for evaluating the effect of pulsatile release profiles, which it is known can improve treatments in conventional formats (**Figure** **9**A,B). The biphasic gels described contain a step in MNP concentration, formed by polymerization in the presence of a field‐gradient generated by a magnet, with a surface field of 0.65 T. This improved deformation and release, even at small gel dimensions, as well as providing a cell‐supporting particle‐free layer.^[^ ^79^ ^]^ Rotating static‐field stimulation was used to achieve pulsatile delivery of mitoxantrone (2 min release at 1 Hz every 2 h, Figure 9A), or of cells (2 min release at 1 Hz every 24 h, Figure 9B). More recently pulsatile release (by exposing samples to 0 and 0.56 T at piston minimum and maximum positions generated using handheld permanent magnets) of mitoxantrone from 8 mm diameter × 2 mm height cylindrical biphasic ferrogels (with MNP‐laden and porous alginate regions) was also studied for melanoma treatment. The effects of drug/pulse number, frequency (1 or 10 Hz), duration and intensity (release rate, measured at 0.25, 2, and 6 h or constant delivery) were evaluated to partially optimize the delivery profile.^[^ ^80^ ^]^ Benefits were demonstrated for treatment of melanoma cells (in vitro) at fixed total mitoxantrone dose. Other groups have investigated delayed delivery for controlled osteodifferentiation of bone morphogenetic factor 2 from biphasic Bulk‐Mag‐Gels upon timed strain‐induced “squeezing,” using similar permanent magnets of up to 0.56 T.^[^ ^81^ ^]^ Optimization of the pulsing and timing for treatments of this type provides great possibilities but is arduous; requiring development of highly stable platforms and automation.

### 3.4 Directional Mechanical Hardening and Electro‐Conductivity

**Figure 10:**
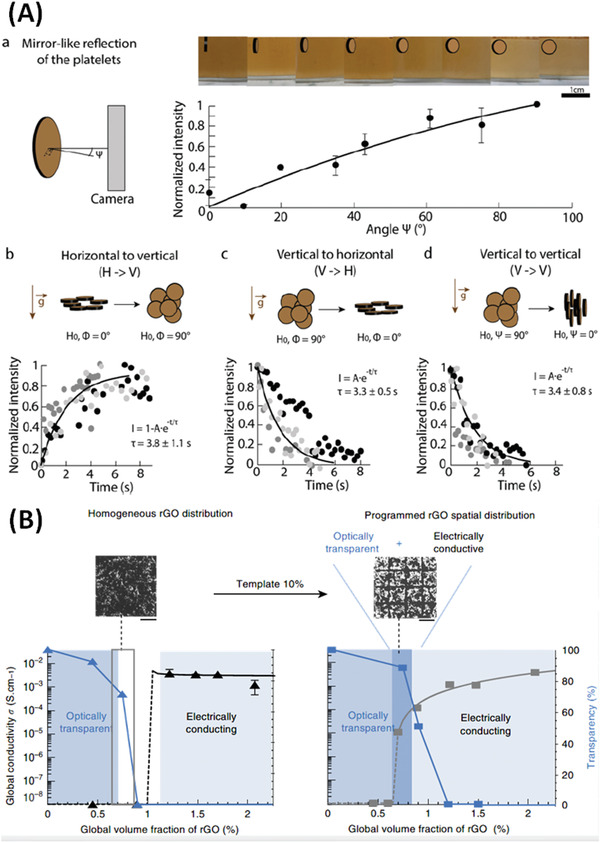
A) Suspension reflectance measurements used to assess the time required for platelet alignment. a) Normalized reflected intensity and corresponding optical micrographs as a function of the platelets’ angle, c. The black line corresponds to the theoretical fittings.^[^ ^46^ ^]^ The optical images show the color change in suspensions containing 25 vol% platelets in 5 wt% PVP aqueous solution subjected to a 165 mT (neodymium) magnetic field rotating at 1 Hz. b–d) Platelet alignment dynamics when the suspension is subjected to a 90°‐step change in the direction of the applied rotating magnetic field. Reproduced with permission.^[^ ^46^ ^]^ Copyright 2019, The Royal Society of Chemistry. (B) Control over rGO spatial distribution using a magnetic template over 10% of area lead to transparent and electrically conductive gelatin films (dark blue region, right) of otherwise opaque and insulating homogeneous films, for total rGO 0.65–0.85 vol% (grey framed region, left). Optical micrographs were obtained from gelatin films containing 0.75 vol% rGO. Scale bar, 500 mm. Reproduced under the terms of a Creative Commons Attribution 4.0 International License.^[^ ^42^ ^]^ Copyright 2016, The Authors, published by Springer Nature.

Le Ferrand et al.^[^ ^46^ ^]^ described a simple drying process for formation of composites with hierarchical internal structures, called magnetically assisted slip casting, a type of magnetic hydrogelation. Alumina platelets were decorated with MNPs and embedded in low‐viscosity dispersions of poly(methacrylate) and polyvinylpyrrolidone, although, in principle many solidifying/gelling polymers could be used. Hierarchical nanocomposites were formed by controlled application of a rotating magnetic field (using a 0.3 T neodymium magnet mounted on an electrical motor) combined with in situ “jamming,” that is, locking the platelets position in a given volume, and finally drying (Figure 4C). The use of rotating magnetic fields allows selection of any, or multiple, precise internal platelet orientation within the nanocomposites (**Figure** **10**A). In a similar approach from Erb et al.,^[^ ^82^ ^]^ visualized in Figure 10A, platelet orientation is determined by the balance of gravitational, viscous and magnetic forces. Le Ferrand and co‐workers also demonstrated temperature‐induced organization of MNP‐coupled graphene oxide (GO) flakes (with interactions mediated by physical links provided by protein bovine serum albumin) embedded within thermoresponsive gelatin (Figure 4D).^[^ ^42^ ^]^ This method allowed flake alignment, on application of a 0.25 T neodymium magnet during gelation and simultaneous graphene oxide reduction, which provided combined optical and electrical responsiveness (Figure 10B). Similarly, aligned Bulk‐Mag‐Gels were formed in a 10 T static‐field (not specified, but presumably an NMR magnet) using MNP‐functionalized GO in an acryl monomer solution, with cross‐linking polymerization by *N*,*N*_0_‐methylenebis(acrylamide).^[^ ^42^ ^]^ Alignment was quantified by SAXS and anisotropic electroconductivity was demonstrated with enhancement along the aligned reduced graphene oxide network component direction (Figure 10B).

MNPs, or MNPs, bound to (diamagnetic) nano‐ or micro‐scale platelets can be manipulated using static‐fields to modulate the internal organization of (bio)polymer networks, enabling control over the mechanical performance of the final solidified/gelled materials. For example An and co‐workers evaluated the potential for magnetic hydrogels (fabricated using magnetic hydrogelation in a 20 mT field of soft physically self‐assembling block copolymers in the presence of MNPs) for enhanced hardening.^[^ ^43^ ^]^ Using magneto‐rheological measurements they showed up to 60 times higher storage modulus for gels with alignment perpendicular to the shear direction, than for random, non‐aligned, gels.

As noted above magnetic hydrogelation is not necessarily confined to the total volume of the sample. In fact, Conte et al. prepared concentrated MNPs surface‐functionalized with thermolysin or chymotrypsin for locally catalyzing transformation of Fmoc‐capped di‐ and tri‐peptides solutions into gels. Hydrogelation in the presence of a static field (neodymium cube, 0.49 T), was used to spatially define locations within the peptide solutions where high local junction concentration (extent of hydrogelation) was specified. This provided up to ≈10‐fold increase in the bulk mechanical storage modulus of the hydrogel as compared to a conventional soluble enzyme system.^[^ ^62^ ^]^

### 3.5 Directional Delivery of Topographical Cues for Tissue Engineering

**Figure 11:**
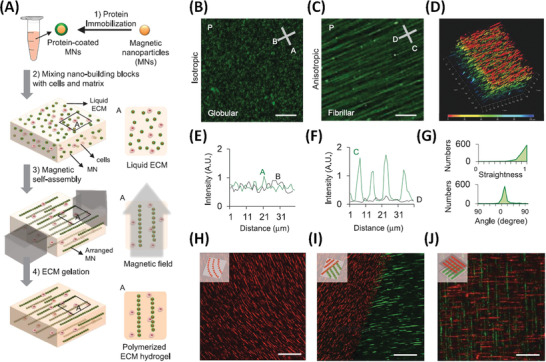
Self‐assembly of functionalized magnetic particles creates diverse topographies in 3D. A) Schematic of the fabrication process. B,E) Isotropic topography composed of randomly dispersed particles coated with green fluorescent fibronectin. C,F) Anisotropic (fibril) topography composed of self‐assembled particles. D) Fibril topography in 3D. G) The straightness (upper graph) and the orientation (lower graph) of nanofibers in the anisotropic topography. H) Curved nanofibers fabricated by the application of a curved magnetic field. I) Two adjoining topographies with different surface proteins and orientations (red: laminin, green: fibronectin). J) Two stacked topographies with different surface proteins and orientations. Scale bars, 30 µm for (B)–(D) and 100 µm for (H)–(J). Reproduced with permission.^[^ ^45^ ^]^ Copyright 2015, Wiley‐VCH GmbH.

Inducing structural (e.g., polymeric fibres/scaffolding) alignment as a means to providing local architectural cues with directional preference has long been a goal in tissue engineering, as an early step toward multi‐scale anisotropic organization of regenerative tissue.^[^ ^83^ ^]^ Magnetically‐enabled approaches can facilitate architectural and biochemical patterning (on the molecular to nano‐scales) of cues within scaffolds. Kim et al. described a magnetic hydrogelation method for directed self‐assembly in a 3D matrix using a neodymium magnet, with subsequent solidification to provide directional alignment of fibres/matrix formers and directional delivery of biochemical cues within the scaffold (**Figure** **11**A).^[^ ^45^ ^]^ Protein coated‐MNP chains (≈5 µm width) were assembled over neodymium magnets to generate alignment within pre‐formulations that were finally gelled (Matrigel and hyaluronic acid) to maintain the structurally programmed chain topography (Figure 11B–G). Anisotropic organization of cultured cells on parallel fibres was confirmed, based on dendrite extension of NIH 3T3 fibroblasts and PC12 cells.^[^ ^45^ ^]^ This approach is not limited to a single type of protein‐bound MNP with parallel orientations, but it can be used to form multiply‐patterned constructs such as curved fibres (Figure 11H), co‐existing domains of patterned proteins within a layer (Figure 11I), or layers with successive perpendicular fiber orientations each with different protein tags (Figure 11J). The process appears to be scalable, low‐cost, and capable of providing spatial organization with nano‐to micro‐scale resolution. Also cells were found to align along the fiber direction, irrespective of the ECM composition, suggesting that topography can override biochemical cues. Both of these aspects are highly encouraging for the development of structured artificial tissue.

MNP chains can also be tagged covalently with bioactive molecules, thus offering opportunities for linear patterning of biochemical cues within the matrix. For example Araújo‐Custódio et al. attached MNPs to cellulose nanocrystals and embedded these in enzymatically cross‐linked gelatin under static‐field stimulus (neodymium, ≈0.1 T) to form distinct anisotropic structures across different length scales, whose organization resembled the hierarchical structure of tendon.^[^ ^84^ ^]^ Vertical alignment of particle chains was also induced (using an unspecified static‐field, presumably a permanent magnet) in polyacrylamide hydrogels to form platforms for rapid development of multicellular spheroids.^[^ ^45^ ^]^ The MNP chains were aligned perpendicular to the hydrogel (outer dimensions 0.2 × 1.0 × 1.0 cm) surface, within wells (of 4, 8, and 12 µm^2^) that had been homogeneously loaded with different MNP concentrations (0.05, 0.3, and 1.8 mg mL^−1^ Fe, respectively). Using this platform, the density of perpendicularly aligned chains in the loaded regions was controlled. Together the anti‐cell‐adhesive nature of the hydrogel, and the spatially organized cell‐adhesive nature of the magnetic colloidal components, forced spheroid formation for both normal and cancerous cells.^[^ ^45^ ^]^

As demonstrated above, magnetic hydrogelation offers a relatively facile way to provide anisotropic constructs that better mimic many native tissues than do conventional hydrogels. In the short term, this is one of the simplest approaches to rapid biomanufacturing of user‐specified anisotropic constructs, requiring MNP dispersion and the use of hand‐held permanent magnets. Modifications that provide additional complexity include; coupling MNPs to different objects (platelets, proteins, rods); layer by layer curing (which we classified in Section 2.3.2.), or; the use of different biopolymers for generating chemically diverse and heterogeneous matrices.

### 3.6 Directional Magnetic Assembly of Cellular Constructs

Micro‐Mag‐Gels can also be used as building blocks of more complex structures, even into the millimeter‐scale. One such example from materials science was described by Xu et al., who developed MNP‐GelMA microgels (≈200–1400 µm size range) by a micro molding technique, where a layer of microgels is hydrogelated by UV‐curing.^[^ ^85^ ^]^ The microgels were organized from a random distribution into rows of chains, using parallel neodymium magnet arrays (retained in position by poly(methyl methacrylate) spacers). The materials were then fabricated into multi‐layered structures by sequentially rotating the magnets by 90° to the base of the chamber and repeating the procedure for up to three steps (in this case). Even more interestingly multi‐layered modular spherical hydrogels, of 1–4 mm diameter were fabricated using three microgel sizes (200, 400, and 1000 µm), composed of three individually gelled layers of different particles.^[^ ^85^ ^]^ This imaginative outcome was achieved by straightforward self‐assembly of Micro‐Mag‐Gels around magnetic rods, which made embedding different functionalities in different layers/shells within the constructs possible. These could be used for temporal programming of biological signals, or for building‐up constructs from different cell types of attached to/embedded in the microgels. The idea of using magnetized biological building blocks, for example, cells, was also suggested.

**Figure 12:**
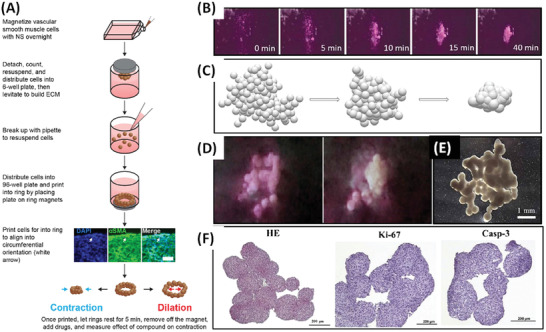
Magnetic assembly for exemplar vascular smooth muscle cells. A) Cells were first magnetized overnight then detached, counted, re‐suspended, and distributed into a 6‐well plate to magnetically levitate in microgravity (space) for 2 h to build ECM. The cells were then re‐suspended and distributed into a 96‐well plate which was then placed on a magnetic drive of 96 ring magnets that assemble the cells into a 3D ring. This aligns the cells into a circumferential orientation as suggested by the alignment of *α* SMA. Subsequently drugs were added to each well and contraction on removal of the field (and loss of the ring) was imaged and measured. Reproduced under the terms of a Creative Commons Attribution 4.0 International License.^[^ ^86^ ^]^ Copyright 2016, The Authors, published by Springer Nature. B–E) Morphological studies of 3D tissue construct obtained by magnetic levitation in microgravity. B) Time‐lapse photographs of the construct assembly inside the magnetic bio‐assembler in microgravity. C) Simulation of chondrosphere fusion into a 3D construct. D) Sequential steps (from time‐lapse video recording) of construct bioassembly in microgravity. E) The assembled 3D construct on return to earth. F) Histology (hematoxylin and eosin staining) and immunohistochemistry (proliferation marker Ki‐67 and apoptosis marker caspase‐3) of 3D tissue construct assembled in microgravity. Reproduced under the terms of a Creative Commons Attribution 4.0 International License.^[^ ^91^ ^]^ Copyright 2020, The Authors, some rights reserved; exclusive licensee American Association for the Advancement of Science.

In the emerging field of magnetic assembly of cellular constructs, MNPs can be uptaken by, or attached to, cells to provide additional magnetic responses in a freeform (self‐) assembly format (**Figure** **12**A).^[^ ^86^ ^]^ In recent reports functionalization was by electrostatic and non‐specific attachment of nanoparticles to the cell membrane via poly‐l‐lysine.^[^ ^86^, ^87^ ^]^ The cells were then printed/deposited onto a base on top of a 96‐well magnetic drive, consisting of 96 neodymium magnets, and field‐gradients were used to drive assembly into cellular spheroids. The same group used magnets to position individual cells into well‐defined spheroids^[^ ^87^ ^]^ and into rings (Figure 12A).^[^ ^86^ ^]^ These methods are particularly useful for rapid prototyping of cellular constructs (e.g., spheroidal models) and organoids used for screening cellular processes such as induced apoptosis (anticancer drug delivery) or differentiation (growth factor delivery).^[^ ^88^ ^]^ Furthermore, the Demirci group demonstrated that non‐magnetically tagged single cells could be magnetically levitated in a paramagnetic medium (using a static‐field gradient of 600 T m^−1^ between two parallel magnets)^[^ ^89^ ^]^ and in this way biological entities could be assembled using magnetic levitation into functional constructs within hydrogels.^[^ ^90^ ^]^ These levitation platforms enable (very high sensitivity) density measurement, imaging, and profiling of cells in real‐time at single‐cell resolution,^[^ ^89^ ^]^ providing possibilities for density‐based sorting or formation of constructs with layers of different density that could subsequently be magnetically hydrogelated.^[^ ^90^ ^]^ Related approaches to magnetic levitation have been commercialized.

Finally magnetic levitational bio‐assembly in microgravity, an exotic example of magnetic levitation assembly, was described for printing assembled cellular constructs in low Earth orbit.^[^ ^91^ ^]^ Magnetic attraction between MNP‐labelled cells (in the absence of gravitational forces, and directed using neodymium magnets) was utilized to form complex 3D chondrocyte spheroids (chondrospheres) and retain their shape over a period of 40 min (Figure 12B–F). The assembled chondrospheres were then “fixed” using NIPAM‐PEG‐based thermoreversible hydrogel, gelling upon increase of temperature to physiological, enabling the constructs to be safely returned to earth for analysis. This experiment conducted on‐board the International Space Station demonstrated formation of usable tissue spheroids; analysis revealed that the chondrocytes maintained their viability and showed typical physiological activity for 3D cultures.^[^ ^91^ ^]^

Additive manufacturing of next‐generation biomedical materials in space has been demonstrated for plastics and small daily‐use objects.^[^ ^92^ ^]^ Studies in microgravity may provide spherical cell constructs to larger sizes, with more rapid tissue assembly than is possible on earth. Such studies may in time provide insights relevant to the work of earth‐bound tissue engineers, as well as, solutions for future travelers.^[^ ^93^ ^]^ The “terrestrial” approaches may eventually contribute to organ regeneration, more immediately their development is likely to support biomanufacturing of organoids for personalized treatment and as more advanced models for aging‐related diseases such as osteoarthritis.^[^ ^93^ ^]^

### 3.7 Shaping Responsive Scaffolds for Tissue Engineering and Regenerative Medicine

**Figure 13:**
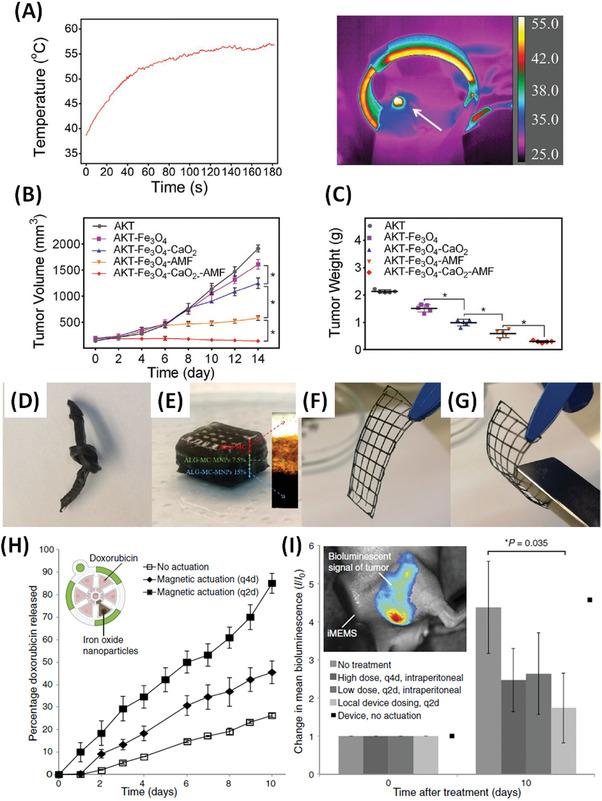
A–C) In vivo synergistic therapeutic performance of the AKT‐MNP‐CaO_2_ composite scaffolds. A) AC‐field heating and infrared bitmap of an AKT‐MNP‐CaO_2_ scaffold implanted in the tumor during irradiation. B) Time‐dependent tumor‐volume of MNNG/HOS bearing mice in different treatment groups (*n* = 5, *p < 0.05). C) The weight of excised tumor from each group after 14 d of treatment (*n* = 5, *p < 0.05). A–C) Reproduced with permission.^[^ ^4^ ^]^ Copyright 2019, Wiley‐VCH GmbH. Demonstration of Mag‐Gel: D) Mechanical stability/knotting; E) MNP concentration gradient within the print (inset, optical confirmation of layers); F,G) Static‐field response, thin Mag‐Gel tiles can be bent, folded and translated. D–G) Reproduced with permission.^[^ ^63^ ^]^ Copyright 2020, Elsevier. H,I) iMEMS as a means for localized, low dose chemotherapy for osteosarcoma. H) In vitro release of Dox from a single‐gear iMEMS device. Schematic diagram is shown in the inset. Dox release was evaluated from devices (*n* = 3) that were not actuated, actuated once every 4 days (q4d), and actuated once every other day (q2d). I) Change in tumor bioluminescent signal over time in a mouse osteosarcoma model treated with Dox. Nude mice injected with luc‐2–transfected osteosarcoma cells developed tumors that produced a bioluminescent signal in the presence of luciferin (inset). H,I) Reproduced with permission.^[^ ^58^ ^]^ Copyright 2017, American Association for the Advancement of Science.

A key advantage of 3D printing for tissue engineering is the relatively facile fabrication of custom‐designed scaffolds in the macro sized range. Dong et al. 3D extrusion‐printed multicomponent akermanite scaffolds (initially in 12 wt% PVA hydrogels) using a nanocomposite ink with co‐loaded calcium peroxide (CaO_2_) and MNPs.^[^ ^63^ ^]^ Synergistic therapeutic effects were described for an in vivo mouse model of osteosarcoma arising from; i) AC‐field hyperthermia; ii) hyperthermically‐enhanced Fenton‐like reactions producing toxic hydroxyl radicals, and; iii) induced bone regeneration due to release of Ca^2+^ from the scaffold. The advantages of printing lay in the macro‐porosity (in the ≈400 µm range for the bone tumor application) and the possibilities of matching xenograft shape (in this case with printed implant size of 6 × 2 × 2 mm). The implanted scaffold (**Figure** **13**A–C) and was shown to retain sufficient rapid AC‐field induced heating, of ≈18 °C over 180 s irradiation at 500 kHz. It was shown that the combined treatment of a scaffold including CaO_2_ and AC‐field exposure was most effective in stopping tumor growth (Figure 13B,C), leading to overall 91.4% inhibition efficacy, compared to 63.2% for scaffold + AC‐field treatment, without CaO_2_.

In other work, it was shown that the potential deleterious effects of radicals generated by MNPs were mitigated by using MNPs doped with carbon quantum dots, an approach that could be readily transferred to other nanocomposites, if required.^[^ ^94^ ^]^ In this case extrusion printing of gelatin nanocomposites in grid‐like patterns was shown to control osteogenic and chondrogenic differentiation (evaluated by histology) in an in vivo rat model. The dual functionality of the particles enabled fluorescent and MR imaging of the printed scaffolds in vivo. Printed scaffolds (1 cm^2^ area, 1 mm height) with Wharton's‐jelly derived mesenchymal stem cells (MSCs) were also magnetically “actuated” (i.e., aligned using a static‐field of 0.05 T, generated by two parallel neodymium magnets) and then subcutaneously implanted under static‐field exposure (to retain orientation of the printed structure with respect to the defect). Initial in vitro experiments demonstrated upregulation of bone‐ and cartilage‐specific gene markers, suggesting responsive induction of the endochondral ossification differentiation route due to localized stress on exposure to the gradient. In this highly promising study, the details of magnetic actuation were regrettably brief and non‐magnetic controls were not described.

Using a different approach, Podstawczyk et al. reported magnetically responsive actuators (structures that responded by bending in the static‐field gradient generated by a 0.23 T neodymium magnet) made of flexible magnetic alginate‐methyl cellulose nanocomposite (Figure 13D–G).^[^ ^63^ ^]^ The nanocomposite inks provided sufficiently viscous mixtures for extrusion printing high fidelity structures (physically supported by a shear‐thinning methyl cellulose component), and were crosslinked after fabrication by immersion in Ca^2+^ and subsequently washed to provide stable structures (a sodium alginate scaffold component). The authors presented possibilities for printing MNP concentration gradients, by applying field‐gradients to the viscous formulation in a cartridge, effectively pre‐organizing particles into chains and generating a gradient prior to printing/crosslinking. 2D‐grid (in xy) structures of ≈1 × 1 × 1 cm, with a vertical z‐gradient of MNP content from 15 to 7.5 and to 0 wt% in different layers were also demonstrated (Figure 13E). Although biocompatibility was claimed, and all the components are known to be biocompatible, the cellular response of the printed structures was not described. Nevertheless, this approach could provide responsive scaffolds for tissue engineering with tuneable inter‐layer mechanical properties and actuation on the sub centimeter‐scale by external fields. Spangenberg and co‐workers 3D extrusion‐printed similar magnetic structures based on a blend of alginate and methylcellulose polymers, into which magnetite microparticles were incorporated.^[^ ^95^ ^]^ In this study two component prints (formed using magnetic and non‐magnetic materials, in separate printheads) were generated, with layers of the magnetic component aligned either perpendicular or parallel to the printing direction, to provide spatial localization of the magnetic part. The gradients (c.10 mT m^−1^) used while printing were achieved with moveable permanent magnets positioned above and below the sample. Cytocompatibility of the constructs was demonstrated post‐printing for the cell‐free scaffolds; i) indirectly by culturing immortalized human MSCs expressing human telomerase reverse transcriptase (hTERT) in supernatants taken from the scaffolds, and; ii) directly by placing hTERT‐MSCs between free spaces in the printed grids, where they showed 74 ± 5% viability on day 21 for printed Mag‐Gels, compared to 82 ± 3% for non‐magnetic bulk gels. These studies highlight significant advantages of conventional 3D extrusion‐based printing of Mag‐Gels, in that they provide; i) custom‐shaped macro‐scale objects that can fit individual defects with selected micro‐porosity, and; ii) possibilities for fabrication of non‐magnetic and magnetic components into scaffolds for providing spatially‐specified responses.

In vivo delivery of fluorescently‐labelled beads, as well as Dox on demand for osteosarcoma chemotherapy treatment from magnetically layer‐by‐layer manufactured devices was demonstrated by Chin et al. (Figure 13H,I).^[^ ^58^ ^]^ Static‐fields were used as a facile delivery switch in vivo (see inset to Figure 13H), offering disruptive approaches to implantable systems (an iMEMS device) with switchable on‐off non‐contact controlled release characteristics. Bioluminescence intensities (normalized to the value before treatment) in an in vivo athymic male nude mice tumor model were compared for four different groups; i) no treatment (control); ii) high systemic Dox dose (administered every 4 days); iii) low systemic Dox dose (every 2 days), and; iv) low local Dox doses administered using the implanted iMEMS device (actuated every 2 days). Systemic doses resulted in a 44% inhibitory rate compared to control, while magnetically actuated delivery resulted in a 60% inhibitory rate, the highest among all treatment groups (Figure 13I). This example demonstrates the potential of implantable magnetic nanocomposite devices for use in personalised therapies, with triggering release by clinicians remotely monitoring patients’ vitals.

### 3.8 3D‐Printed Soft Magnetic Robots

**Figure 14:**
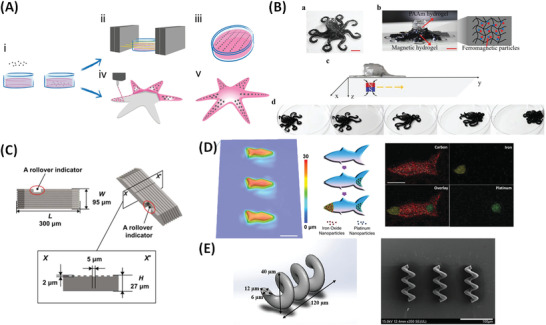
A) Schematic representation of the anisotropic nanocomposite fabrication and the 3D‐printed star‐shaped magnetic soft robot. i) MNP addition to a liquid precursor suspension at >37 °C. ii) Application of low‐intensity static‐field. iii) Formation of oriented MNP chains on cooling induced hydrogelation. iv) 3D printing of the Mag‐Gel formulation onto a hydrogel substrate. v) UV‐crosslinking resulting in a stable star‐shaped responsive structure. Reproduced with permission.^[^ ^7^ ^]^Copyright 2019, Wiley‐VCH GmbH. B) a) 3D printing of soft octopus robot. b) Front view of the robot which is fabricated in two parts; i) Acrylamide‐Carbomer ink is used to print the transparent head; ii) MNP‐loaded acrylamide‐Carbomer ink is used to print the tentacles to provide responsive propulsion shown schematically in (c); and in (d) programmed left to right propulsion is confirmed. (Scale bars, 1 mm.) Reproduced with permission.^[^ ^53^ ^]^ Copyright 2019, Wiley‐VCH GmbH. C) Computer‐aided design images and dimensions of the Micro‐Mag‐Gel robots used for neural networks. Reproduced with permission.^[^ ^98^ ^]^ Copyright 2020, The Authors, some rights reserved; exclusive licensee American Association for the Advancement of Science. No claim to original U.S. Government Works. Distributed under a Creative Commons Attribution Non Commercial License 4.0 (CC BY‐NC. D) 3D microscopy image of an array of microfish, scale bar 100 µm, fabricated by a layer‐by‐layer continuous optical printing. Schematic illustration of the process of functionalizing a microfish for guided catalytic propulsion, see text. Spatial localization of Pt nanoparticles in the tail of the fish (propulsion) and MNPs in the head (orientation) confirmed by EDX, see text. Scale bar, 50 µm. Reproduced with permission.^[^ ^63^ ^]^ Copyright 2015, Wiley‐VCH GmbH. E) Fabrication of a helical robot and SEM confirmation of its structure. Reproduced with permission.^[^ ^63^ ^]^ Copyright 2019, Wiley‐VCH GmbH.

Although there are recent reviews that include descriptions of the additional capabilities of magnetic additive manufacturing in the context of 4D printing and soft magnetic robots,^[^ ^9^, ^12^ ^]^ none to date describe in detail the types of magnetic manufacturing and link internal architecture of fabricated constructs to their final applicability. Magnetic manufacturing techniques have been used to produce free‐swimming robots of different dimensions from millimeter down into even the low micron‐scale that can respond to applied fields. At the upper end of this size range, Ajiteru et al. used DLP to prepare functional Mag‐Gel bioreactors on the centimeter‐scale.^[^ ^96^ ^]^ These structures were capable of inducing rapid (within 2 s) strain in encapsulated non‐magnetic materials, leading up to uniaxial 2 mm/33% strain extension, in a cyclic and reproducible manner on exposure to static‐field gradients generated by two axially arranged cylindrical neodymium magnets (0.3 T), driven toward each other (cyclically) using a stepped motor. Karshalev et al. fabricated functional centimeter‐scale magnetic fish.^[^ ^97^ ^]^ Platinum NPs were embedded in the “tails” during screen printing of this component to provide “self‐propulsion” by catalytic reaction of H_2_O_2_ “fuel” with gaseous O_2_ expelled. Incorporation of magnetic Nd_2_Fe_14_B microparticles in printed strips provided capability for autonomous re‐orientation. Self‐healing was also facilitated, re‐establishing the swimming action for fish that had been cut at different positions. Augurio et al. printed (slightly smaller) centimeter‐scale magnetically responsive nanocomposite scaffolds using luminescent inks that incorporated core‐shell SrF_2_ upconverting NPs in gelatin methacrylate.^[^ ^7^ ^]^ MNP suspensions were then backfilled into the prints and the MNPs were aligned into chains using static‐fields (generated by two neodymium magnets separated by 5 cm, generating a static 0.02 T) prior to UV‐curing. These multi‐responsive mobile platforms (with multi‐scale structuring) have potential for real‐time deep monitoring (emissive nanocomponent) and remote manipulation (magnetic nanocomponent). Furthermore, Tognato et al. combined both magnetic manufacturing and, as described in Section 2.1, magnetic hydrogelation, to fabricate a series of stimuli‐responsive scaffolds with internally aligned architecture (**Figure** **14**A).^[^ ^7^ ^]^ Spherical MNPs were incorporated into gelatin methacrylate solution which was cast as a warm suspension, with gelation on cooling with or without static‐fields (using the same setup of two magnets at 5 cm). Further, the authors described the use of extrusion‐based 3D printing to generate anisotropic geometries by embedding MNPs in the extremities of the printed structures. These soft robotic coin‐sized “stars,” of ≈22 mm diameter, had magnetically responsive arms and non‐magnetic cores whose movement in the presence of field‐gradients (generated by a set of three electromagnets placed either on the left, the right, or at the bottom of the water pool) was demonstrated.

Chen et al. fabricated millimeter‐scale magnetic robots by extrusion‐based 3D printing using chemical crosslinking of polyacrylamide and carbomer colloids with MNPs (Figure 14B).^[^ ^53^ ^]^ The resulting soft “octopi” were capable of movement following the field‐gradients generated by a moving neodymium magnet. Other millimeter‐scale devices included sugar‐based helical robots 3D printed by selective laser sintering.^[^ ^99^ ^]^ Gervasoni and co‐workers demonstrated that by controlling laser power, and so extent of caramelisation, the architecture and mechanical properties of the sugar‐based structures could be controlled. By incorporating MNPs in the helical structures, millimeter‐scale helical swimmers capable of corkscrew motion in a rotating magnetic field (30 mT, 5 Hz) were demonstrated.

Spatiotemporally controlled electrodeposition is a flexible manufacturing method that can be used to form many types of magnetically responsive structures, including magnetic robots. Although this technique is not really a fabrication approach, in the sense described here for hydrogels, due to its versatility and the possibility of generating hydrogelation encapsulated electrodeposited structures we present some interesting examples here. Hu et al. used double template‐assisted electrodeposition of porous magnetic microstructures,^[^ ^100^ ^]^ in an approach that combined 2D photolithography and electrophoretic assembly of polystyrene beads to form a confined micron‐scale mold space for controlled electrochemical growth of porous magnetic cobalt–nickel alloy. The porous magnetic microstructures were loaded with dye‐loaded alginate hydrogels to form field‐gradient controllable microtransporters. Layered Mag‐Gel structures can also be prepared using conventional UV photocuring and masks. In one such study, Kim et al. formed intraocular injectable dissolvable microrobots, with bilayer (biphasic) magnetic structures for AC‐field induced (*ν*_AC_ = 272 kHz, *H*_AC_ not specified, *t* = 6 min) Dox delivery, which also have potential for subsequent retrieval using field‐gradients generated by an eight‐coil electromagnetic actuator system (capable of generating 45 mT and gradients of up to 8 mT m^−1^).^[^ ^101^ ^]^

Considering smaller length scales, Kim et al. fabricated 300 µm × 95 µm magnetic microrobots with a groove‐pattern (2 µm height × 5 µm width) using two‐photon polymerization‐based 3D laser lithography which were capable of inducing extended parallel axonal outgrowth (Figure 14C).^[^ ^98^ ^]^ Using field‐gradients (generated again by an eight‐coil system, in this case of up to 20 mT, with 2 T m^−1^) neuron‐ and MNP‐loaded microrobots were positioned at gaps between clusters of neuronal cells. These were cultured on a metal‐oxide semiconductor‐based high‐density multi‐electrode array, designed to allow tracking of any propagation of extracellular axonal signals. Confocal microscopy, neurite extension and electrophysiological signals confirmed successful bridging of the neuronal clusters. At the lower end of currently accessible sizes, Zhu et al. used micron‐scale continuous optical printing to rapidly fabricate multiple, 30 µm × 120 µm, micro‐swimmers, or “microfish” (Figure 14D) from polyethylene glycol diacrylate (PEGDA).^[^ ^63^ ^]^ Similarly to the report described above,^[^ ^97^ ^]^ platinum NPs were embedded in the “tails” post printing to provide catalytic “self‐propulsion” using H_2_O_2_ “fuel,” and MNPs were embedded in the “heads” (Figure 14D) to provide static‐field responsive orientation to the propulsion. Furthermore, polydiacetylene nanoparticles were also incorporated into the microfish to provide catalytically mobile agents to extract the toxin melittin from aqueous fuel doped environments and to prove that the approach could in principle be extended to other relevant biomolecules. Magnetic PEGDA microrobots, on a similar length scale of 40 µm × 120 µm, were developed for delivery of 5‐fluorouracil (5‐FU) mediated by controlled degradation.^[^ ^63^ ^]^ Helical microrobots were fabricated from PEGDA and pentaerythritol triacrylate with MNPs and pre‐loaded with 5‐FU, by 2‐photon polymerization (Figure 14E). Slowly rotating field‐gradients (11 Hz, 24 mT) were again generated using an eight‐coil system that provided stable (due to the helical structure) directional robot movement. Local AC‐field induced 5‐FU release was shown, providing potential utility for cancer treatment as suggested by in vitro drug‐release/HCT116 (human colorectal cancer) cell studies. The polymer degradation rate was controlled (through curing exposure, copolymer formulation, and PEGDA molecular weight) and tuned to ≈30 h total time, providing a background release rate which could be accelerated with an exposure‐dependent response by application of the AC‐field. Interestingly, no temperature increase was observed for the helices, although this formulation has a strong response (∆*T* ≈ 20 °C after 10 min at 430 kHz, 45 kA m^−1^) in bulk. Hence the localized AC‐field induced increase in 5‐FU diffusivity within the helices is clear. In contrast, Dong et al. formed magnetoelectric helices of the order of 50–100 µm (in length) manufactured from GelMA and MNPs consisting of a CoFe_2_O_4_ core and a BiFeO_3_ shell.^[^ ^102^ ^]^ The “magnetoelectric” helices were used as attachment sites for the differentiation and delivery of SH­SY5Y neuronal like‐cells, and generated electric fields upon exposure to an AC‐field (39.78 kA m^−1^, 1.05 kHz).

Finally, populations of near‐identical magnetic micro‐ and nano‐robots can in principle be fabricated at scale, enabling combined or cohort tactics, for example, for localized delivery of therapeutics and tracking (typically by MRI, but also perhaps in time by MPI, or even dual opto‐magnetic modalities). Cohorts in this sense are inspired by nature and typically referred to as “robotic swarms.” Wang and Zhang recently reviewed the capabilities of such swarms and described the inter‐robot interactions that give rise to coordinated movement.^[^ ^103^ ^]^ Unlike non‐isotropic MNP‐loaded robots controlled by synthetic procedures,^[^ ^104^ ^]^ 3D light processing technologies enable control over shape in large robot numbers, so this is the more likely approach for manufacturing swarms of nominally identical, multifunctional magnetically‐responsive robots. A more detailed account of the trends in micro‐ and nano‐robot design (not limited to magnetic responders) can be found in the recent review by Wang and colleagues.^[^ ^105^ ^]^

### 3.9 Achieving Hierarchically Complex and Responsive Structures

The most structurally and functionally complex structures achieved to date using magnetic fabrication methodologies come from the magnetic extrusion‐based printing or magnetic stereolithography. These include millimeter‐scale objects that can be formed from components that embed nanometer‐scale spatial organization.

**Figure 15:**
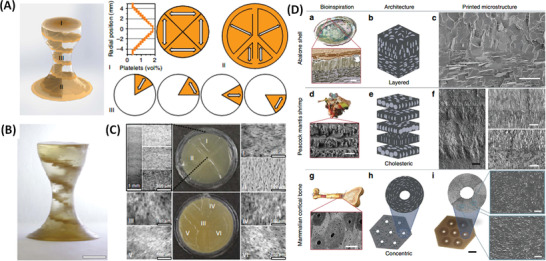
A) Design and programming of the heterogeneous composite. B) Actual MM‐3D printed object with internal helicoidal staircase. Scale bar, 5 mm. C) Photograph of the top layer of the structure confirming locally variable platelet alignment. A–C) Reproduced under the terms of a Creative Commons Attribution 4.0 International License.^[^ ^56^ ^]^ Copyright 2016, The Authors, published by Springer Nature. D) Examples of bioinspired microstructured composites. a) The Haliotidae sp. Abalone shell exhibits a layered structure of calcite prisms topping in‐plane aragonite platelets (nacre). Reproduced with permission.^[^ ^106^ ^]^ Copyright 2014, Wiley‐VCH GmbH. This architecture is b) simplified and c) 3D magnetic printed. d) The peacock mantis shrimp dactyl club exhibits a cholesteric architecture of mineralized chitin fibres. Reproduced with permission.^[^ ^107^ ^]^ Copyright 2014, Elsevier. This architecture is e) simplified and f) 3D magnetic printed. g) The mammalian cortical bone exhibits concentric plywood structures of lamellae‐reinforced osteons.^[^ ^108^ ^]^ Reproduced with permission.^[^ ^109^ ^]^ Copyright 2006, Elsevier. This architecture is h) simplified and i) 3D magnetically printed. All printed microstructures are acrylateurethane co‐polymers reinforced by 15 vol% alumina platelets. Scale bars (mm), a) 5; c) 25; d) 15; f) 50 (black) and 20 (white); g) 200; i) 5 (black), and; 25 (white). Reproduced under the terms of a Creative Commons Attribution 4.0 International Licens.^[^ ^57^ ^]^ Copyright 2015, The Authors, published by Springer Nature.

In the example by Kokkinis et al.,^[^ ^56^ ^]^ described in Section 2.3.3, by controlling the rotation of the neodymium magnet (40 mT at 8.3 Hz) during printing, magnetized alumina platelets were formed into complex internal architectures including helical millimeter‐scale staircases (**Figure** **15**A–C).^[^ ^56^ ^]^ These structures better recapitulate the internal ordering and complex architectures of tissues, and so may prove in time to be a suitable approach for advanced tissue engineering applications. In another example, Martin et al. used magnetic stereolithography to create highly programmable discontinuous fibres inspired by the biologically relevant structures of abalone shells, peacock mantis shrimp, and mammalian cortical bone (Figure 15D).^[^ ^57^ ^]^ This method allowed formation of materials with superior spatial control of the exact orientation of the magnetized particles, with similar organization to osteon microstructures with concentric reinforcement orientation and “monolithic” parts, providing a benchmark for improved mimics of native tissues.

In a recent example of magnetic stereolithography Li et al.^[^ ^64^ ^]^ incorporated micro‐bundles of MNPs aligned in the printing direction using a magnetic ring approach, a simpler version of the configuration shown in Figure 3C. The resulting enhanced compression resistance and anisotropic mechanical integrity enabled preparation of limpet tooth‐inspired polymer microneedle arrays, with microneedle sizes reported with resolution as good as 10 µm. Conical tooth‐like structures with apex angle of as small as 15° and total diameter of base of 50 µm were also developed. The magnetic microneedle arrays were found to generate transient holes in mouse dermal tissue, and to release Rhodamine B in vitro and Fluorescein into porcine skin ex vivo. Finally, structures that respond with spatiotemporal control to fields by means of controllable programming of magnetization encoded during magnetic extrusion‐based printing can be generated.^[^ ^59^, ^110^ ^]^ In one example, Zhu et al. fabricated a set of functional centimeter‐ to millimeter‐scale soft materials, including crawling robots, flexible grippers, bionic butterflies, and multistate magnetic switches for which reconfiguration of their movement was possible using a static magnetic field (neodymium, ≈0.20 T).^[^ ^110^ ^]^ This magnetic fabrication approach allows combination of programmed magnetization (and thus response), and in principle can also be combined with magnetic hydrogelation/additive manufacturing to provide internal alignment leading to complex architectures.

## 4 Conclusions, Challenges, and Future Perspectives

The fabrication of magnetic nanocomposites for biomedical and tissue engineering applications has evolved rapidly in the last two decades; multi‐scale hierarchical organization and field‐responsiveness of these materials can spatially encode and trigger physical, chemical and biological signals and cues. The programmed responses arise from; i) the type of network, classified by the intrinsic cohesive/repulsive particle‐polymer molecular interactions and the type of chemical bonds/network crosslinks, which in turn dictate the final topology/properties, and; ii) the stimulus responses of the magnetic components, mediated in the nanocomposites by inter‐MNP interactions (dispersion vs chain formation) or by the loading and concentration of magnetized microscale particles (including anisotropic platelets or rods). Control over these aspects has been shown to provide network stiffening or remote actuation on application of static‐fields or static‐field gradients, or controlled hyperthermically‐induced network swelling or transition on application of AC‐fields (when thermosensitive bonds or polymers are included). The possibilities for realizing these responses in tissue engineering are only beginning to be realized.

An evolving set of magnetic fabrication methods are being used today to prepare bioinspired composites in custom‐designed patterns with control over a wide range of length scales. The scales involved for the responsive components range from 10 to 100 nm (for MNPs), to 0.5–50 µm (for magnetized microparticles or cells), to 100–1000 µm for printed features or selectively loaded voxels/locations. It has been established that simultaneous independent control over the organization on each of these length scales is possible with fidelity across the entire printed object. Inclusion of MNPs during magnetic extrusion‐based printing provides (particle and fiber) alignment opportunities, which can also arise in the absence of fields due to strong dipolar interactions mediated by particle shape or by geometric constraints (e.g., from shear fields during printing). This promising area is not well explored, for instance the extent of alignment, and hence of directional magnetic properties, is not generally maximized.

The temporal part of the encoded response arises both from the timing of the stimulus (or stimuli, as these can be temporally complex, pulsed, etc.) and the objects response to the stimulus. Temporal control over actuation has been reported, for example in centimeter‐scale bioreactors,^[^ ^96^ ^]^ and the systems are usually shown to be stable to repeated action. However stability to extensive cycling (100–1000 times) is not described and responses are typically slow, which may limit translational possibilities. Precise control over the stages of deformation or positioning on short time scales is a persistent challenge for conventional hydrogels, so magnetically responsive systems may usefully accelerate both deformation/actuation and provide precise positioning.^[^ ^26^, ^55^, ^101^ ^]^

While fabrication with multi‐scale internal organization is technically challenging, some of these possibilities have already been realized, at least in part, with multiple examples highlighted here of; i) patterning of bulk‐ and micro‐structures, including concentric, layered, and lamellar hierarchical systems, providing patterned distortion, heating, stiffening, and release, and; ii) functional free‐swimming robots (including stars, octopi, and helices), fabricated with high precision across a wide range of length scales providing directed chemical function and motion. We suggest that, while functional patterned materials will in time have many biomedical applications, promising devices (of the two types noted) that may be close to realization include patterned non‐contact actuation/cell stimulation systems (for automated growth of organoids, spheroids, or cell clusters), and robots with non‐contact external magnetic navigation (for remotely‐targeted delivery).

Significant technical challenges remain with magnetic additive manufacturing processes, and to date no such technology has been made generally available. Hence the wide range of exciting options for field control and structuring (described in Section 1.3, Figure 3) are yet to be fully exploited. Perhaps this is to be expected, given the early stage of development of the subject with many printing modes (Sections 2.3.1–2.3.4) still under investigation and no commercial devices currently available. Progress would be accelerated both by availability of such instruments, and by addressing the inconsistency in the details (printhead speeds, magnet positioning, etc.) that are reported. Together the community should require that sufficient technological detail be provided in publications, to minimize the need for repetition/re‐development. This deficiency makes performance comparisons of the final nanocomposites difficult, exacerbating the underlying problem of designing reproducible, up‐scaled magnetic inks.

Materials issues that arise include batch‐to‐batch variability of the MNPs intrinsic magnetic properties, a persistent difficulty rarely acknowledged in the literature. Ensuring colloidal stability and hence homogeneity of magnetic inks from run‐to‐run (and from batch‐to‐batch), both before and during printing, is also critical and can be challenging. For instance AC‐field hyperthermia applications require efficient heating; for suspensions this necessitates good MNP dispersion (random inter‐MNP dipolar interactions reduce SAR), or formation of short MNP‐chains (oriented inter‐MNP dipolar interactions can enhance SAR).^[^ ^111^ ^]^ However, there are only a few studies evaluating the aggregation state of MNPs in inks or printed constructs.^[^ ^23^ ^]^ Applications that exploit particle alignment require sufficient MNP mobility within the matrix formulation for MNP chains to form, or for microparticles to orient, in the field. Good cohesion between the MNPs and network is also necessary to avoid particle leaching, or uneven structural response; both aligned MNP‐chains and magnetized microparticles may fit this purpose. There is also the need for magnetic inks with shear‐thinning and recovery sufficient for extrusion, which can be crosslinked after printing by multiple physicochemical means. Formulations that address these exhaustive criteria for any given magnetic additive manufacturing mode (layer‐by‐layer, extrusion, and stereolithographic) are scarce, and it is highly unlikely that a single formulation will prove suitable for multiple modes. The current situation has given rise to a huge complexity and diversity of new inks, and so there is a pressing need for generally applicable formulation rules for magnetic additive manufacturing, there has been some progress in this direction.^[^ ^112^ ^]^

Finally it is not currently possible to use conventional bio‐inks in magnetic additive manufacturing to generate complex structures, so that avenue is open for development. On the other hand the magnetic hydrogels that are currently used present complex handling and biocompatibility issues, including long‐term bio‐stability. Although the fact that MNPs have been FDA‐approved for clinical MRI suggests that including the magnetic functionality may not complicate this issue. For clinical translation in tissue engineering fabrication of constructs that, even more faithfully, mimic native tissue organization and have programmed responses (e.g., spatiotemporally‐controlled in vivo cue delivery) is needed for implants which match heterogeneous and hierarchical tissue types, including spinal cord,^[^ ^113^ ^]^ bone,^[^ ^114^ ^]^ cartilage,^[^ ^115^ ^]^ and muscle.^[^ ^116^ ^]^ Given the advances reported here, with standardization of printing modes and with advanced magnetic inks formulated using approved components, significant progress in this direction may be possible in the medium term. Inclusion of orthogonal techniques, for example, the application of additional external stimuli such as acoustic fields^[^ ^117^ ^]^ may assist in generating the necessary ordering in more complex scenarios. The ultimate goal should be to push these challenges away from materials science and into the specialized tissue engineering laboratories.

## Conflict of Interest

The authors declare no conflict of interest.
